# Ferroelectric triggering of carbon monoxide adsorption on lead zirco-titanate (001) surfaces

**DOI:** 10.1038/srep35301

**Published:** 2016-10-14

**Authors:** Liviu Cristian Tănase, Nicoleta Georgiana Apostol, Laura Elena Abramiuc, Cristian Alexandru Tache, Luminița Hrib, Lucian Trupină, Lucian Pintilie, Cristian Mihail Teodorescu

**Affiliations:** 1 National Institute of Materials Physics, Atomistilor 405A, 077125 Măgurele-Ilfov, Romania; 2 University of Bucharest, Faculty of Physics, Atomiştilor 405, 077125 Măgurele-Ilfov, Romania; 3 University of Trieste, Department of Physics, Via Valerio 2 - 34127 Trieste, Italy

## Abstract

Atomically clean lead zirco-titanate PbZr_0.2_Ti_0.8_O_3_ (001) layers exhibit a polarization oriented inwards P^(−)^, visible by a band bending of all core levels towards lower binding energies, whereas *as introduced* layers exhibit P^(+)^ polarization under air or in ultrahigh vacuum. The magnitude of the inwards polarization decreases when the temperature is increased at 700 K. CO adsorption on P^(−)^ polarized surfaces saturates at about one quarter of a monolayer of carbon, and occurs in both molecular (oxidized) and dissociated (reduced) states of carbon, with a large majority of reduced state. The sticking of CO on the surface in ultrahigh vacuum is found to be directly related to the P^(−)^ polarization state of the surface. A simple electrostatic mechanism is proposed to explain these dissociation processes and the sticking of carbon on P^(−)^ polarized areas. Carbon desorbs also when the surface is irradiated with soft X-rays. Carbon desorption when the polarization is lost proceeds most probably in form of CO_2_. Upon carbon desorption cycles, the ferroelectric surface is depleted in oxygen and at some point reverses its polarization, owing to electrons provided by oxygen vacancies which are able to screen the depolarization field produced by positive fixed charges at the surface.

Apart for their validated applications in piezoelectronics, pyroelectric materials, high permittivity dielectrics, light valves^1^, and non-volatile memories^2^, ferroelectrics were recently promoted as valuable candidates for catalysis^3^, photocatalysis^4^,^5^ and solar cells^5^,^6^. Owing to the band bending occurring at surfaces of the ferroelectric layers exhibiting out-of-plane polarization, outwards (P^(+)^) polarized faces are active for reduction, and inwards (P^(−)^) polarized faces are active for oxidation^7^,^8^. Ferroelectricity in a ‘perfect’ (i.e. insulating and insulated from ground) layer with no contaminants is possible only *via* the formation of ferroelectric (mostly 180°) domains, otherwise the depolarization field due to bound charges, oriented anti-parallel to the polarization, is sufficient to destroy the polarized state^9^. However, single domain ferroelectric ultrathin layers are routinely synthesized nowadays, using advanced deposition techniques, such as pulsed laser deposition (PLD)^8^,^10^,^11^,^12^,^13^, up to thicknesses of three lattice constants (about 1.2 nm^14^,^15^). This is due to the presence of some other charges in the system, able to compensate the depolarization field. These charges may be located in the metal electrodes for metal-ferroelectric-metal (MFM) sandwich structures, may be due to adsorbates from environmental atmosphere^16^, charge carriers injected from the metal inside the ferroelectric semiconductor, or may originate from donor- or acceptor-like defects in the semiconductor^9^. According to a simple model where the depolarization field is compensated by sheets of mobile charge carriers located at a depth *δ* under each surface, the band bending is expressed as *eP*_⊥_*δ*/∈, where *e* is the elementary charge, *P*_⊥_ the out-of-plane component of the polarization and ∈ the dielectric permittivity^10^,^11^. Introducing usual values for high quality crystalline ferroelectric thin films of lead zirco-titanate (PZT)^11^, *P*_⊥_ ≈ 0.4 C/m^2^, *δ* ≈ 3 nm, and ∈_*r*_ ≈ 180, this yields a band bending of 0.76 eV (These values, estimated one decade ago will be re-evaluated later for the actual samples.). Recently, band bendings even larger (in the range of 1 eV) were reported for pulsed laser deposited PZT^12^,^13^, consistent with a higher value of the polarization^17^, approaching 1 C/m^2^. Another approach is to attribute the origin of compensating charges to adsorbates or ionic surface reconstructions, a passivation mechanism found more stable thermodynamically than the compensation by mobile carriers^16^,^18^. In this case, the orientation of the surface electric field and the band bending at surface is opposite with respect to the compensation by accumulation of mobile charges below the surface. Indeed, ad-anions such as O^2−^ or OH^−^ are shown to stabilize P^(+)^ states, whereas ad-protons stabilize P^(−)^ states^15^. All the above considerations lead to the natural conclusion that there is a delicate interplay between the polarization state, the ability of the semiconductor to provide charge carriers to compensate the depolarization field, the role of the charge which may be transferred by the adsorbates, and the catalytic properties of the ferroelectric surfaces^5^,^18^.

A second aspect worth to be mentioned refers to the combination between organic molecules and ferroelectrics. Self-assembled monolayers (SAMs) of alkylsiloxanes synthesized on sol-gel prepared PZT were found to increase the corrosion protection (to HCl-containing media) of the ferroelectric^19^, however the characterization by XPS in this early work did not evidence any polarization-dependent component, but just Zr and O components corresponding to hydroxylated oxide species lying underneath the SAMs. More recently, BaTiO_3_ nanocrystals modified by n-hexylphosphonic acid have shown considerable improved dielectric properties^20^ but, again, no consideration on the ferroelectricity of the substrate in influencing chemical bonding with the adlayers was mentioned. The reverse was postulated, namely that the stabilization of tridentate bonding with deprotonated phosphonate yields to considerable lower losses, most probably due to the organic surrounding of nanocrystals, acting as an insulating loss barrier which reduces inelastic scattering of charge carriers^20^. Hence, associating organic molecules with ferroelectrics leads to the fabrication of novel materials with improved properties. One should therefore try to study in deeper detail the role of the ferroelectricity in such associations.

Amongst the catalytic reactions of main interest nowadays, oxidation of carbon monoxide is of prime importance^21^. Exhaust gas motors contain also NOx complexes together with unburned fuels, therefore nowadays the ‘three way catalysts’ must favor both reduction (of NOx) and oxidation (of hydrocarbons and carbon monoxide) reactions. This is usually achieved by using nanoparticles formed by precious metals (Pt, Pd, Rh), but research is still performed in order to find cheaper and more efficient materials, operating at lower temperatures. Ferroelectric materials are good candidates for such applications implying both oxidation and reduction processes, and catalysts may be developed using these bare materials or combined with metal nanoparticles. For instance, it was shown that a high efficiency CO oxidation may be achieved on PbTiO_3_ nanoplatelets (np) and on Pt/np-PbTiO_3_; in the latter case, a 100% conversion is attained at temperatures below 370 K^22^. However, no direct connection was established between the ferroelectric state and the catalytic efficiency. More recently, it was reported that selective deposition of Pt on P^(+)^ faces of PbTiO_3_ yielded one order of magnitude better efficiency in H_2_ generation by photocatalytic water splitting^23^, therefore P^(+)^ faces are efficient for reduction processes. Back to carbon monoxide, *ab initio* computations have shown clearly that CO adsorption is strongly favored on P^(−)^ faces of PbTiO_3_ with 1 monolayer (ML) Pt on top^3^,^24^. Dissociative chemisorption of carbon monoxide occurs for P^(−)^ faces only^24^. In view of these computations, the catalytic and photocatalytic activity would be explained by the nature of the bonds at the surface, whereas other models involve charge transfer mechanisms from the bulk towards the surface^25^,^26^,^27^. For instance, it was supposed that near the ferroelectric to paraelectric (FE → PE) phase transition, the elevated value of the dielectric constant yields to the increase of the Debye length (∈*k*_B_*T*/*N*_*D,A*_)^1/2^/*e*, where *k*_B_*T* is the Boltzmann factor and *N*_*D,A*_ the density of donors or acceptors in the space charge region; thus, photogenerated charges produced in the bulk of the ferroelectric are field extracted from a thicker region and transported towards the surface to promote oxidations or reductions^26^,^27^. Thin metal or oxide overlayers do not influence much this polarization-induced chemical or photochemical activity^27^. Polarization specific experiments are reported since a few years only, such as the reduction of Ag^+^ on P^(+)^ areas together with Pb^2+^ oxidation to Pb^4+^ on P^(−)^ areas of BaTiO_3_^27^, reduction of Ag from AgNO_3_ on P^(+)^ areas of PZT^28^,^29^, faster desorption of CO_2_ from P^(−)^ areas of BaTiO_3_ and PZT^30^, similar results with stronger desorption of simple organic polar molecules from P^(−)^ faces of KNbO_3_^27^ (also strongly enhanced at the FE → PE transition^31^) and LiNbO_3_^32^,^33^,^34^,^35^, and the preferential adsorption of polar contaminants on P^(+)^ areas of PZT^36^. If the polarization state of the ferroelectric surface is found to influence strongly the adsorption/desorption kinetics and possibly the surface chemical reactions, the reverse is also valid, i.e. molecular adsorption may yield to reversible polarization switching^37^. In view of the above experimental evidence and of the tremendous number of possible applications, *real* surface science experiments performed on atomically clean, well characterized ferroelectric surfaces are expected to provide essential information on the surface chemistry and of its interplay with the polarization state. For instance, dipoles are formed by adsorbates even on nonpolar surfaces^38^, which renders difficult the interpretation of core level positions near these surface fields, not to speak about the two main mechanisms of screening: the fixed surface charges, either by adsorbates and by ionic reconstructions^5^,^16^,^18^,^32^,^33^,^39^, or by accumulation of mobile carriers near the surface^7^,^8^,^10^,^11^,^12^,^13^,^40^, but inside the material. These two mechanisms imply opposite surface fields, opposite band bendings and hence opposite catalytic activities (transport towards surface of charges of opposite sign). The origin of extrinsic dipole fields due to adsorbates must be ruled out prior to any investigation of adsorption and catalytic properties of these polar surfaces. One needs then to work in ultraclean conditions, on well characterized surfaces from structural and compositional points of view.

By taking care of possible side effects^38^,^41^, X-ray photoelectron spectroscopy (XPS) would be a favorite tool for such investigations, owing to its chemical and surface sensitivity, which was recently converted also in the ability to derive surface band bendings^8^,^12^,^13^,^36^,^40^,^41^,^42^,^43^,^44^. In this work, we show how this feature is combined with the natural ability of XPS to derive states and amounts of molecules adsorbed on surface^45^. The main experimental difficulty is to prepare well defined ferroelectric surfaces. In this work, 20 nm thick, atomically clean PZT(001) surfaces were obtained from PZT films prepared by PLD by a treatment in a well outgassed ultrahigh vacuum (UHV) environment implying annealing at about 700 K for 3–6 hours, following ref. 46. Prior to the UHV cleaning procedure, all samples are routinely characterized by X-ray diffraction^43^,^44^, atomic force microscopy (AFM) and piezoresponse force microscopy (PFM)^8^,^12^,^13^,^36^,^44^. After cleaning, PZT(001) samples exhibited good low energy electron diffraction (LEED) patterns. We will unambiguously identify that atomically clean layers present a well defined polarization state, the sign of the band bending correspond to screening of the depolarization field by charges located inside the material (hole accumulation or missing cations) and only in this state the surface is able to adsorb CO, partly dissociating these molecules, at room temperature. The sticking of carbon and of CO on the surface is also found to be related to the non-vanishing out-of-plane polarization state.

## Results and Discussion

### Piezoresponse force microscopy

#### Instability of P^(−)^ states in air

**Figure 1:**
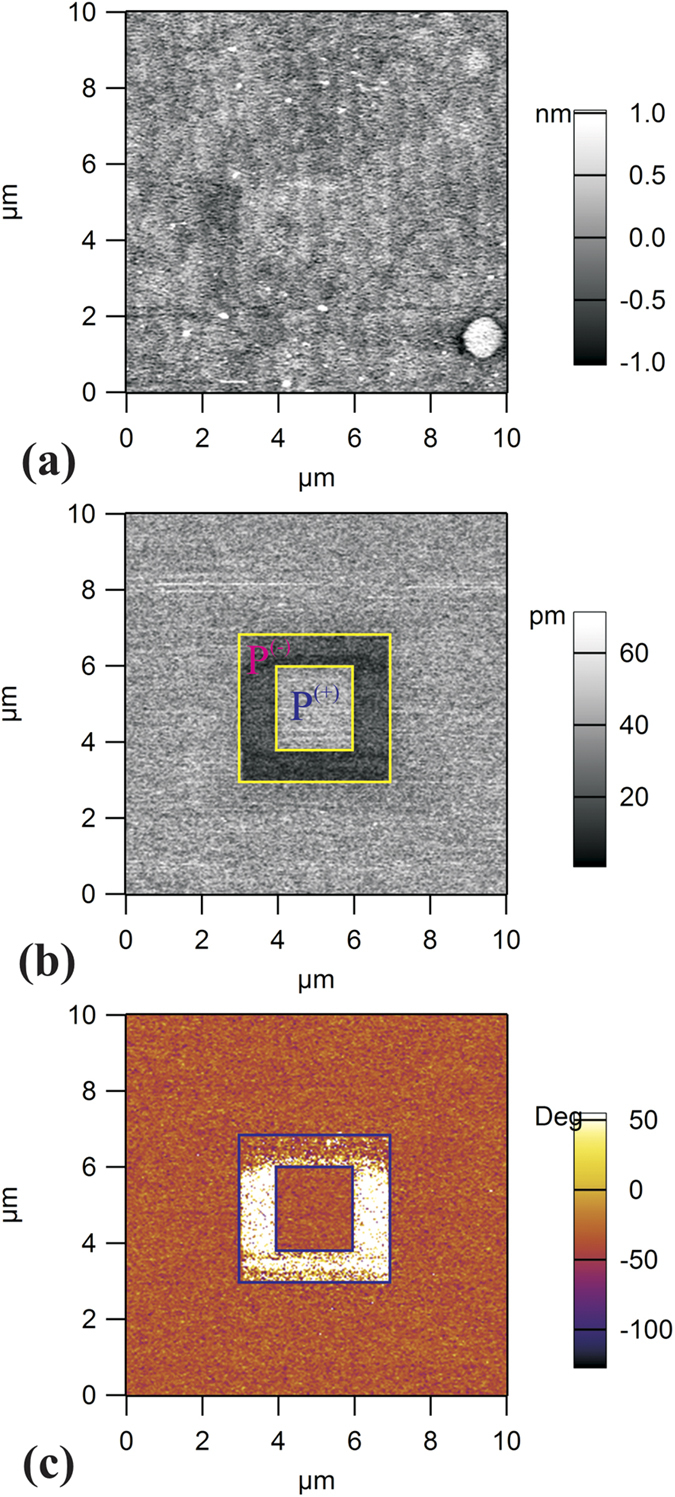
Atomic force microscopy (AFM) and piezoresponse force microscopy (PFM) investigation of a twin PZT sample to the one used in the photoemission experiments (all other Figs): (**a**) topography, AFM signal; (**b**) Amplitude PFM signal after poling, together with the poling map; (**c**) Phase PFM signal, after poling.

Immediately after preparation, all samples are investigated by PFM, as represented in Fig. 1. The phase signal, which is sensitive to the out-of-plane polarization state, did not show any contrast, which is a sign that the whole analyzed surface has a similar out-of-plane polarization. This might be P^(+)^ (outwards) or P^(−)^ (inwards). The layers are then poled, according to a poling map represented also in Fig. 1(b). The AFM conductive tip was set to a positive voltage (+12 V) for P^(−)^ poling and to a negative voltage (−12 V) for P^(+)^ poling. The phase signal, after poling, was the same for the P^(+)^ poled area as for the unpoled areas, which is a sign that the initial polarization state of the whole film was P^(+)^. P^(−)^ states are not stable: they vanish after some tens of minutes, as can be seen from Fig. 1(c), where the P^(−)^ poled area already started to be washed out after about 3–5 minutes from the poling. Comparatively, thicker PZT layers (150 nm) yielded much more stable P^(−)^ areas^13^,^36^. The ability of free surfaces of thicker layers to produce P^(−)^ areas with longer lifetimes can be connected to the ability of these layers to provide positive charge near surface for compensation of the depolarization field. This free charge might be either ionized donors (oxygen vacancies) or holes. Both mechanisms may be proposed at this point to justify the longer lifetime of P^(−)^ states for thicker layers. A thicker layer (~150 nm) with a concentration of defects (acceptors) on the order of 4 × 10^25^ m^−3^ might be able to produce enough holes which, under the influence of the depolarization field, migrate near the surface to form a layer with a charge density numerically close to *P*, on the order of 1 C/m^2^. When the thickness of the layer is decreased by one order of magnitude, simply there might be not enough defects in the film to produce the required mobile charges (mechanism 1).

The other possibility (mechanism 2) is that the positive charge is formed by ionized donors of density *N*_*D*_^(+)^, while electrons migrate towards the bottom electrode. Oxygen vacancies are shallow donors with the corresponding donor levels (*E*_*D*_) situated a few tenths of eV (0.06 or 0.3 eV for the two type of vacancies V_O1_ or V_O2_, respectively) below the bottom of the conduction band (*E*_*C*_)^47^. We consider that the density of oxygen vacancies (*N*_*D*_) exceeds the density of states from the conduction band, which is given by *N*_*C*_ = (2πm*k_B_*T*)^3/2^/*h*^3^ (m* = electron effective mass, *k*_B_ = Boltzmann constant, *T* = temperature, *h* = Planck constant). For m* = 0.09 *m*_*e*_ (*m*_*e*_ = electron’s rest mass) in PZT^48^, one obtains *N*_*C*_ ≈ 6.5 × 10^23^ m^−3^. It is clear that *N*_*D*_ is much larger for the samples synthesized by PLD since (as an estimate) 10% missing oxygen from one formula unit, e.g. a composition such as ABO_2.9_ (occupying an average volume of 64 Å^3^) yields *N*_*D*_ ≈1.6 × 10^27^ m^−3^. Thus, the Fermi level position will be given by^49^:


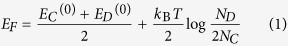


and at room temperature it will be situated at about 0.09 eV above the middle of the distance between the bottom of the conduction band and the donor level. Thus, with a good approximation, the Fermi level can be considered as pinned to *E*_*C*_ (above or below *E*_*C*_ by about 0.06 eV for V_O1_ or V_O2_ vacancies, respectively). Therefore, in the region of the band bending upwards^8^, donors are ionized in a layer of thickness equal to the extent of the band bending *δ*, which is evaluated from 3 to 23 nm for Pb(Zr_0.2_Ti_0.8_)O_3_ and Pb(Zr_0.3_Ti_0.7_)O_3_, respectively^11^. The conclusion is that, for thin layers, the overall thickness becomes comparable with *δ* and therefore less ionized donors are available near the interface. The screening of the depolarization field is not effective and probably holes (mechanism 1) or ionized donors (mechanism 2) are progressively neutralized by electrons from the layer, despite the fact that they are attracted towards the bottom SrRuO_3_ electrode.

Probably, a lower compensation of the depolarization field induce a progressive decrease of the average electric polarization, which in turn induces a lower depolarization field, thus a lower separation of the mobile charges and/or density of ionized donors near the interface, up to the vanishing of the out-of-plane polarization and even to the restoring of the opposite polarization, P^(+)^. This state is more stable simply because it supposes the localization near interface of electrons to compensate the depolarization field, and, owing to the large density of donors, it is expected that electrons are more abundant than holes or than the available density of ionized donors near interface.

It follows also that the polarization state must be supplemented by a detailed analysis of the sample stoichiometry near interface, in order to evaluate important parameters, such as the amount of oxygen vacancies, *N*_*D*_. The phase diagram presented by Highland and coworkers^50^ yielded the stability of the single P^(−)^ phase in low O_2_ pressures (below 1 Pa), an intermediate region presenting 180° domains, and the stable single P^(+)^ phase above 10 Pa. The compensation of the depolarization field is attributed in the above Reference to excess O^2−^ ions adsorbed on the surface for the P^(+)^ state, and to a deficit of O^2−^ on the surface to stabilize the P^(−)^ state, which is quite similar to ‘mechanism 2’ proposed above, relying on ionized oxygen vacancies (a ‘ionized oxygen vacancy’ is just a missing O^2−^ anion). Thus, the low stability in air of the P^(−)^ state may also be viewed as the compensation of ionized oxygen vacancies by oxygen (or other anions) from the environmental atmosphere. It appears therefore important to check the stability of the P^(−)^ state in ultrahigh vacuum environment.

### Cleaning procedure in ultrahigh vacuum, investigated by XPS

**Figure 2:**
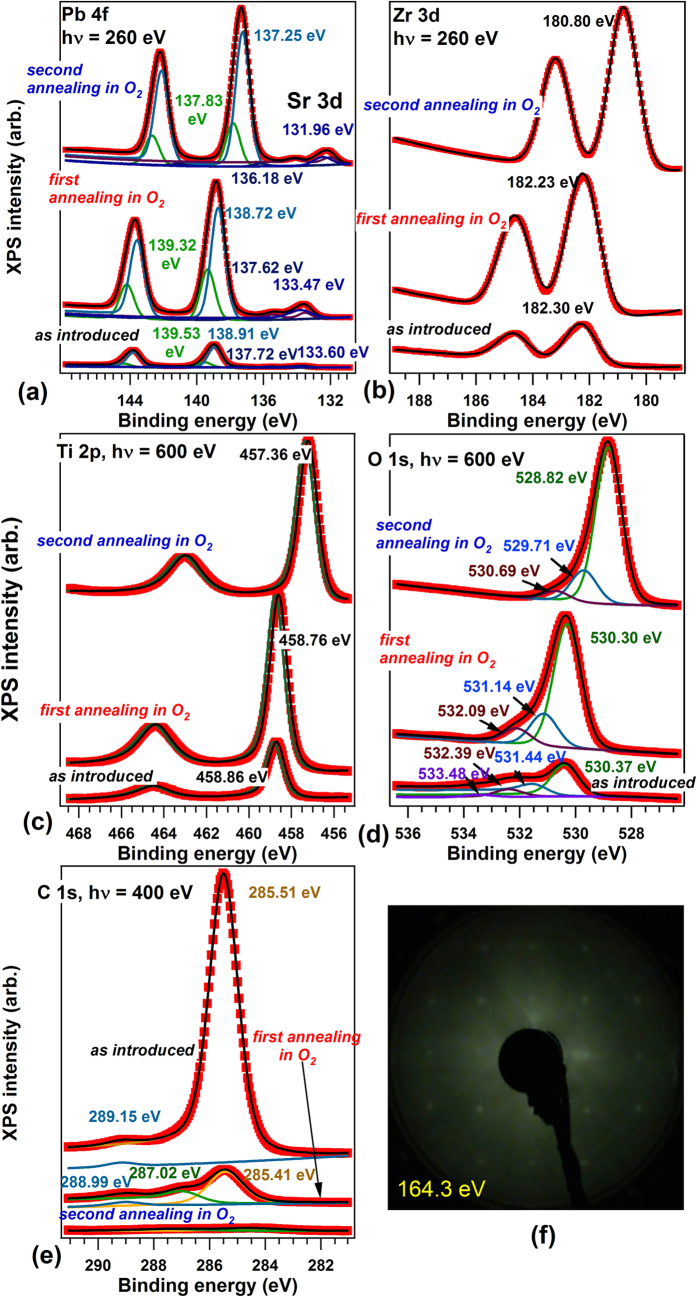
High resolution X-ray photoelectron spectroscopy for an *as introduced* PZT film, after a first annealing procedure (2 h in P[O_2_] = 5 × 10^−5^ mbar), and after a second annealing cycle: (**a**) Pb 4f, photon energy 260 eV; (**b**) Zr 3d, photon energy 260 eV; (**c**) Ti 2p, photon energy 600 eV; (**d**) O 1s, photon energy 600 eV; (**e**) C 1s, photon energy 400 eV; vertical offsets are introduced for clarity; (**f**) the low energy electron diffraction (LEED) pattern obtained after cleaning, electron kinetic energy 165.6 eV.

Figure 2 presents X-ray photoelectron spectra recorded by using synchrotron radiation for the *as introduced* sample, after a first and after a second annealing cycle (3 hours in 5 × 10^−5^ mbar O_2_ pressure). The spectra are simulated (‘deconvoluted’) with Voigt profiles and with Voigt integrals for the inelastic background^51^. More details about the deconvolution procedure, including the formulas used and relations between fitting parameters may be found in ref. 52. The analysis procedure did not take into account eventual variations of the transmission function of the analyzer and photoelectron diffraction effects^53^. Estimates derived for atomic composition are presented in Table 1. Sample #1 is the first sample which was cleaned by two cycles of annealing in O_2_ and whose XPS spectra are represented in Fig. 1. Sample #2 is a sample cleaned once the procedure was well established, and before a cycle of heating and cooling in ultrahigh vacuum, with results which will be discussed in a next paragraph. Sample #3 is a sample prepared before one of the CO adsorptions, which will be discussed towards the end of this Section.

The results of this procedure are binding energies (BE) for individual components and their integral amplitudes. These amplitudes are afterwards renormalized with respect to atomic photoionization cross section^54^ and to the known synchrotron radiation flux (a factor of 2.31 between the flux at the energy of 260 eV, used to record the Pb 4f and Zr 3d spectra, and at 600 eV, where Ti 2p and O 1s spectra are measured). The region of the Pb 4f spectrum includes also a low binding energy feature, which is attributed to Sr 3d mixed with a Pb Auger line (N_7_VV). The Pb 4f spectrum itself was ‘deconvoluted’ with three spin-orbit splits doublets, whereas for Ti 2p and Zr 3d only one doublet suffice to simulate these spectra. The O 1s is deconvoluted with an appropriate number of singlets (the higher binding energy ones being due to contaminants).

The main components of Pb 4f (denoted by Pb_*bulk*_) and O 1s are compared with the whole intensity from Ti 2p and Zr 3d. Pb 4f has also a lower BE component attributed to metal Pb (varying between 2.8% and 2.1% of the main component for Sample 1, 3% of the main component for Sample 3, negligible for Sample 2) and a higher BE component, which might be attributed to PbCO_3_^55^, when combined with the O 1s component at 531.4 eV for the ‘as introduced’ sample. For ‘clean’ samples, the high BE components of Pb 4f (137.8 eV) and O 1s (529.7 eV) signal combines in a stoichiometry such as PbO^12^. Thus, the most correct attribution for these components for clean samples is that they belong to the last PbO layer. Ba 4d photoemission spectra in BaTiO_3_ are interpreted in the same terms^53^,^56^. Let us remind also that the Ti 2p and Zr 3d are well fitted with only one doublet, thus all Ti and Zr atoms are in bulk like environments (or, if some areas were (Zr, Ti)O_2_ terminated, this might not be visible by XPS, see the comments in § 3.5). The fact that such layers are PbO terminated was inferred also for other samples prepared in similar conditions^12^,^13^,^36^,^43^. This Pb 4f component will be denoted by Pb_*surf.*_ in the following, whereas the main component, of highest intensity, was already denoted by Pb_*bulk*_. By taking into account the inelastic mean free path (IMFP) effects and, again, neglecting photoelectron diffraction, the ratio between these two components may be expressed as [Pb_*surf.*_]:[Pb_*bulk*_] = exp{*d*/(*λ*cos *θ*)} − 1, where *d* (~2 Å) is the distance between two layers (or ‘thickness of the surface layer’), *λ* is the IMFP and *θ* the take-off angle (40°). By introducing the value obtained after the first annealing for Sample 1 [Pb_*surf.*_]:[Pb_*bulk*_] = 0.456, one obtains *λ* ≈ 6.9 Å, which is a reasonable value for a kinetic energy of about 121 eV^45^.

The lower values for this ratio obtained after the 2^nd^ annealing (and for Samples 2 and 3) will be discussed a few lines below. Note that the [Zr]:[Zr + Ti] ratio, for all surfaces investigated, is fairly close to the composition of the target used in PLD, PbZr_0.2_Ti_0.8_O_3_. Thus, these ratios seem not to be affected by photoelectron diffraction or different analyzer acceptance. However, the [Pb_*bulk*_]:[Zr + Ti] ratio is always bigger than unity, with the exception of the ‘as introduced’ sample. Here photoelectron diffraction effects may become important. Note also that the final state of the photoelectron has stronger anisotropy for Pb (4f initial states promote electrons in outgoing waves of d and g symmetries, according to the dipole selection rules), whereas outgoing waves of lower angular momenta manifest for Ti 2p and Zr 3d (s and d, respectively p and f). Thus, with all these complications, the amplitude ratios from Table 1 might not be directly used to derive stoichiometries, but rather to infer some main differences between the sample surfaces.

The most striking evidence from Fig. 2 is that, as soon as the surface becomes free of carbon, all core levels shift simultaneously at lower binding energies, by 1.44 ± 0.04 eV. Thus, the clean sample (after the second annealing in O_2_) exhibits P^(−)^ polarization, if one supposes that the preliminary investigations by piezoresponse force microscopy experiments performed in air are still valid. Also, similar samples were used in ref. 13 and the binding energies were similar to that obtained in the actual experiment on the *as introduced* sample. More comments about the binding energies for each polarization state, and a comparison with recent results reported in refs 12, 13 and 36 are detailed in the Electronic Supplementary Information ESI (SI-1). It is therefore reasonable to suppose that the observed shift of 1.44 eV is between P^(+)^ and P^(−)^ state, therefore *eP*_⊥_*δ*/∈ ≈ 0.72 eV. Indeed, the P^(+)^ state requires electrons near surface to screen the depolarization field and these electrons may be provided by the contaminants^18^,^36^,^38^,^50^, by the bottom electrode^43^ or by the oxygen vacancies (the recently reported ‘self-doping’)^44^. When most of the contaminants are removed, Sample 1 after the first annealing, the sample surface exhibit a relatively low [O]:[Zr + Ti] ratio (about 2.3). Thus, oxygen vacancies subsist in the layer to produce the needed electrons for compensating the depolarization field near the surface with outwards polarization (see also Fig. 1 from ref. 8 and Fig. 6 from ref. 44). After the second annealing procedure in oxygen, the oxygen content increases (about 3 per formula unit), together with a decrease of the [Pb_*surf.*_]:[Pb_*bulk*_] ratio. This might be interpreted as the creation of some Pb vacancies in the first surface layer. At the same time, the increase of the [Pb_*bulk*_]:[Zr + Ti] ratio might be interpreted also as the creation of Ti and Zr vacancies. In a similar way oxygen vacancies produce electrons, cation vacancies produce holes to compensate near the surface the depolarization field in the inwards polarized films. Thus, warming up the layer in oxygen atmosphere yields, at a certain moment, oxygen uptake to cancel oxygen vacancies, and the creation of cation vacancies to provide holes for the depolarization charge sheet. The complete removal of any contaminants could somehow be linked to this polarization switch.

The P^(−)^ state is stabilized, as in the case of low oxygen exposure in refs 36 and 50. This result is at variance with the common belief that ultrathin (~10 nm) PZT(001) grown on SrRuO_3_ are stabilized in the P^(+)^ state^29^. This is based on simple considerations of electron injection from the bottom electrode, which has a lower work function (4.7–4.9 eV)^43^; electrons may be injected from the bottom electrode to form the depolarization charge sheet near the outer surface of PZT(001); at the same time, the band bending at the bottom interface corresponds to an electric field oriented towards the surface, which may induce the polarization of the layers near the bottom electrode; then, the poling propagates towards the surface during the film growth. For thicker PZT(001) layers and for insulated thin (20 nm) SrRuO_3_ layers between PZT and the substrate SrTiO_3_, the situation could be more complicated, since electron depletion of the bottom electrode and the development of an electric field at this interface may be limited by the lowering of its work function. (This situation will be detailed in a more extended work.) And, as a final remark, photoholes induced by the photoemission process and subsequent Auger and secondary electron emission may also be used by the film to build up a depolarizing charge layer near surface composed by holes, able to stabilize the P^(−)^ state^8^. In order to check this assertion, and also to quantify possible influences of surface photo-voltaic effects^38^, periodically the beam was switched off from the sample for some tens of minutes, and then we measured again and obtained the same spectra as before switching the beamline off. Another experiment was performed by using an electron flood at several intensities between 0 and 2 mA, yielding very tiny (below 0.02 eV) changes in binding energies. Hence, the PZT layers are rather good semiconductors.

### Temperature dependence of the polarization

**Figure 3:**
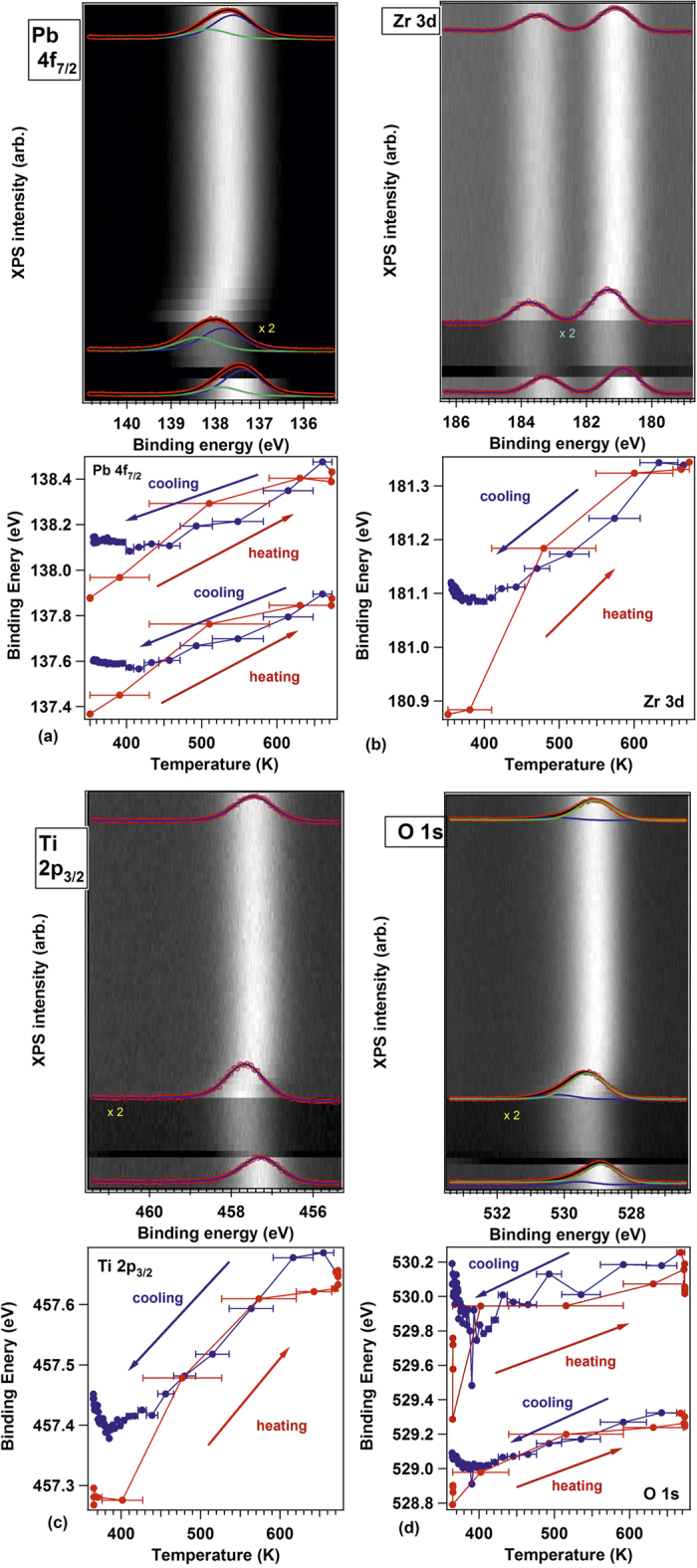
Core level spectroscopy during cycles of heating and cooling. Upper panels: series of XPS spectra, represented on grayscale (bright = largest intensity) with some examples of deconvolutions superposed. Lower panels: evolution of binding energies obtained by deconvolution with the measured temperature. (**a**) Pb 4f_7/2_, photon energy 260 eV; (**b**) Zr 3d, photon energy 260 eV; (**c**) Ti 2p_3/2_, photon energy 600 eV; (**d**) O 1s, photon energy 600 eV.

Up to now, the derivation of the stabilization of P^(−)^ states for ultraclean samples was based only on models where compensating charges are accumulated inside the film and the bands bend upwards near surface. In order to check the validity of this hypothesis, cycles of heating-cooling while measuring XPS were performed. Figure 3 presents the XPS evolution of all core levels with a cycle of heating up to about 700 K (400 °C) and cooling down of the layer. We avoided warming up more, since in other experiments considerable diffusion of Sr from the SrRuO_3_ buffer layer was observed starting with 850 K. The Curie temperature of 20 nm PbTiO_3_/SrRuO_3_(001), based on refs 14 and 15, is of 800–900 K, thus it is expected that by the actual heating cycles the polarization does not vanish completely. The heating-cooling cycle manifest in reversible shifts in BE of all elements by about 0.5 ± 0.1 eV, when all core levels are taken into account. Thus, the P^(−)^ polarization is connected to a band bending upwards, then to a decrease of binding energies for all core levels. Warming up yields to the considerable decrease of the P^(−)^ polarization. The order of magnitude of this shift is reinforcing that the *as introduced* sample had initially (*as introduced*) a P^(+)^ polarization, as derived by PFM and as expected for a contaminated sample able to absorb (mainly hydroxyl) anions as compensating charges.

Note that an irreversible increase of binding energy with temperature was reported for magnetron sputtering prepared PZT/SrRuO_3_/SrTiO_3_(001)^57^ (though without an analysis of sample cleaness nor of its composition). Prior to the photoemission experiments, the film was found P^(+)^ poled. In the view of the actual model employed to explain our results from Fig. 3, the Pb 4f_7/2_ binding energy should have decreased by decreasing the polarization with temperature. However, if one assumes that in the films discussed in Ref. 57 the depolarization field is compensated by the presence of anionic contaminants and not by electrons from the material, the band bending for P^(+)^ is upwards, thus the binding energy decreases due to the contaminants. By warming up, the polarization is lost and also the contaminants are desorbed; at the same time, it may happen that oxygen vacancies (n-type dopants) are created in the layer, and the final state might be with P^(+)^ polarization recovered, but with screening performed by electrons below the surface, with the band bending downwards. Also, the fact that F 1s from BF_3_ exhibited a lower binding energy for adsorption on P^(+)^ areas may be attributed to the formation of compensating charges by adsorption of contaminants (prior to BF_3_ adsorption) with opposite band bending (upwards). The presence of contaminants may be inferred in ref. 57 on LiNbO_3_(0001) single crystals, subject to a similar cleaning procedure as PZT(001); indeed from the XPS survey scans one may estimate, after scaling with atomic sensitivity factors, an atomic ratio C:Nb of about 40%. By taking into account IMFP effects, this implies more than one carbon ad-atom per surface elementary cell. Back to our samples, their cleaness rule out any presence of extrinsic dipoles due to adsorbants; also, there was no reconstruction such as *c*(2 × 2) or (1 × 6) observed^39^. If any surface anion excess would be found, implying a P^(+)^ state with charge compensation located outside the film, by warming up rapidly and assuming, as in refs 32 and 33, that the surface negative charge is not rapidly removed from the surface, one should have observed a considerable decrease of the binding energy with the temperature (as shown by the initial trend for the Pb 4f_7/2_ peak in ref. 57). In our case, the observed and almost reversible increase in binding energy with temperature appears to be relatively independent on the heating rate, since the heating for experiments from Fig. 3 were performed at about 4–5 K/s, the cooling down was considerable slower, while the experiments from the next Subsection were performed at heating rates of about 0.01 K/s. Thus, these measurements confirm a P^(−)^ state of the PZT thin film, with compensation realized from inside the sample, and band bending upwards.

Table 1 presents composition analyses also for this layer, before and after heating. After a heating-cooling cycle, the [O]:[Zr + Ti] and [Pb]:[Zr + Ti] ratios both decrease and also the band bending decreases. This might be interpreted also as an increase of the concentration of B^4+^ cations (Ti^4+^ or Zr^4+^), possibly by diffusion from inner layers, such as less hole generators are needed to compensate a lower polarization. We noticed also an increase of the [P_*bsurf.*_]:[Pb_*bulk*_] ratio, which reinforce the idea that, before heating, the surface PbO layer did not cover entirely the surface.

### Interplay between polarization and CO adsorption

**Figure 4:**
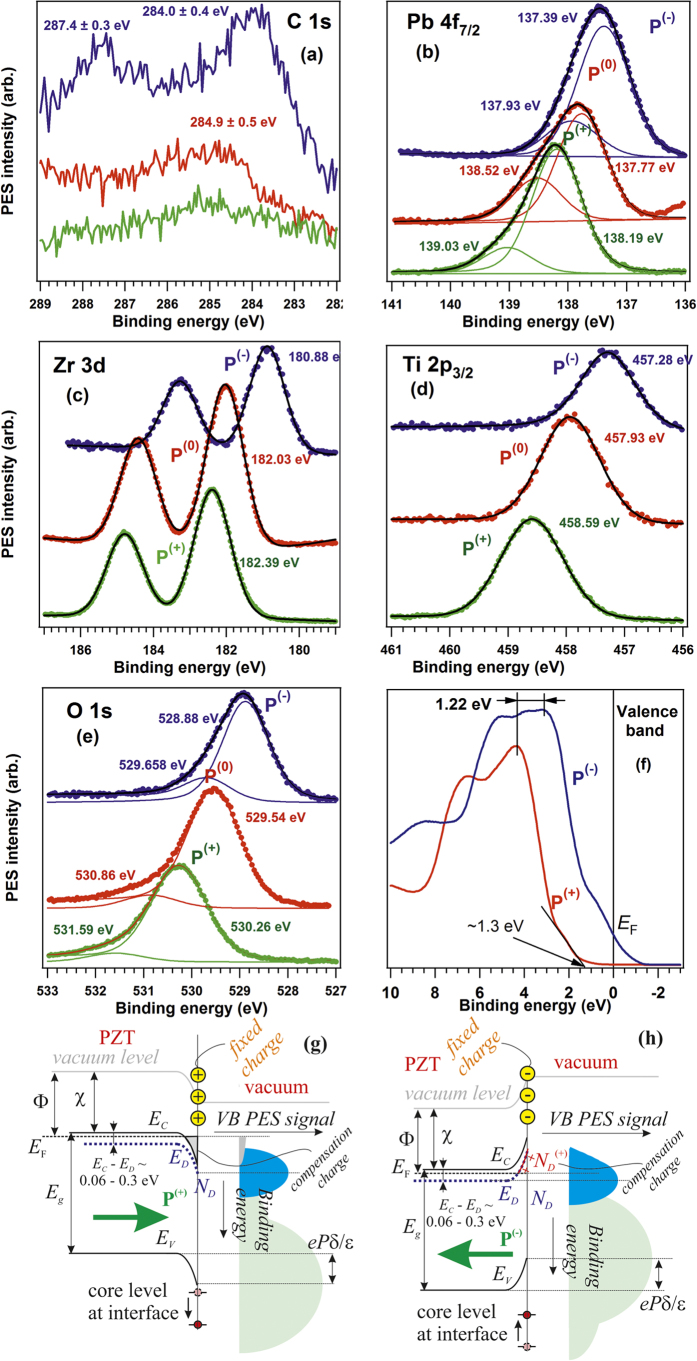
Evidence of the interplay between the initial polarization state and the degree of CO adsorbed on the surface (**a**) C 1s, photon energy 400 eV; (**b**) Pb 4f_7/2_, photon energy 260 eV; (**c**) Zr 3d, photon energy 260 eV (**d**) Ti 2p_3/2_, photon energy 600 eV; (**e**) O 1s, photon energy 600 eV (**f**) valence band, photon energy 100 eV; (**g**,**h**) represent models of interface band diagrams for P^(+)^ and P^(−)^ polarizations. See text for more details.

6000 Langmuir (L) of carbon monoxide (600 s × 1 × 10^−5 ^Torr) were dosed on PZT(001) layers exhibiting both initial P^(+)^ or P^(−)^ polarization, and also one one film with no out-of-plane polarization. The CO amount dosed is close to the saturation found for 2-propanol or acetic acid on LiNbO_3_(0001)^32^,^33^. The results are presented in Fig. 4. The P^(+)^ state was obtained only after one heating cycle of 3 hours (the LEED pattern was good), and the corresponding spectra are represented by green curves. After dosing CO, the C 1s signal increases slightly and, from all other core levels, it seems that the out-of-plane polarization is lost. It seems also that carbon is adsorbed only in reduced form. Most probably, the oxygen vacancies are compensated by oxygen from CO, thus some carbon remains at the surface (estimated to be at most 10% of a monolayer, by ‘monolayer’ understanding one carbon atom per surface elementary cell, about 16 Å^2^). For the loss of polarization, an outline such as that sketched in ref. 12 may be proposed, in the sense that the surface becomes conductive due to this low amount of carbon, and charges cannot be accumulated anymore on this surface, thus the band bending vanishes. Adsorption of other polar contaminants on freshly prepared PZT(001) layers yielded a similar effect^36^.

Contrary to the above case, in the case of an initial P^(−)^ state, a considerably higher C 1s signal is obtained, with two clear components, one corresponding to reduced carbon (binding energy of about 284 eV) and the other one corresponding to C=O (binding energy of about 287.4 eV)^58^. Thus, CO is adsorbed in both dissociated and molecular state on PZT(001) with initial P^(−)^ polarization.

Figure 4(f) represents valence band measurements, together with a sketch of the assertions from the first paragraph concerning band bending, the position of the Fermi level, the ionized donors and the electrons accumulated near the surface as possible source of compensation of the depolarization field for the P^(−)^ and P^(+)^ cases, respectively. The limited angular resolution of the analyzer in the mode used in this experiment guarantees that more than a Brillouin zone is investigated (in fact, more than 1 Å^−3^) by using photon energy of 100 eV. Then, the valence band will be used as a picture of the density of states. For the P^(+)^ state, the onset of the photoemission signal occurs at about 1.3 eV below the Fermi level, whereas for the P^(−)^ state there is a non-negligible signal also at the Fermi level. From a large number of valence band spectra recorded with different setups on PLD prepared PZT(001) layers, we were *never* able to achieve an onset of the photoemission signal close to the value of the bandgap (about 3.4 eV) of the PZT. This should have been the case when, as stated in previously, the Fermi level were almost pinned to the bottom of the conduction band, therefore the valence band signal should occur at a binding energy close to the bandgap for samples with no out-of-plane polarization P^(0)^, and at even higher binding energies for samples with P^(+)^ polarization, owing to the band bending downwards near surface. For instance, in ref. 12, about 1.4 eV was obtained for this onset of the valence band signal. It was then stated in the Ref. 12 that the layer is slightly p doped, since the Fermi level was believed to be closer to the valence band than to the conduction band.

In the following, we will use the derived inequality that for moderate deviations of the oxygen stoichiometry, the density of donors exceeds by several orders of magnitude the density of states in the conduction band and perhaps also in the valence band, i.e. *N*_*D*_ ≫ *N*_*C*_, *N*_*V*_. For P^(+)^ polarization, bands are shifted downwards and near the surface the Fermi level crosses both the donor levels *E*_*D*_ and the bottom of the conduction band *E*_*C*_. The latter crossing has as an effect the availability of this region to accumulate electrons, which are screening the depolarization field. The crossing of *E*_*D*_ has as a consequence the fact that donor levels near the surface will be completely filed. Then, by taking into account also the huge *N*_*D*_, a photoemission experiment will detect a weak signal at the Fermi level due to the accumulated electrons near the surface, and a strong signal at a binding energy equal to (*E*_*C*_ − *E*_*D*_) + *ePδ*/∈, i.e. the difference between the conduction band and the donor level plus the band bending. By using the values derived in the present work, this exceeds 1 eV, if one takes for granted the values for *E*_*D*_ computed in ref. 59 for PbTiO_3_. Note that if one supposes that *E*_*C*_ − *E*_*D*_ ≈ 0.6 eV, a better match with the actual data, and also with the valence band spectra from ref. 12 are obtained (in that case, the band bending was higher than in the actual case by about 0.14 eV and also the onset of the valence band was derived at 1.38 eV binding energy, instead of 1.3 eV).

For the P^(−)^ areas, the band bending occurs upwards. This time the donor levels near surface are depleted, but in principle the occupied levels near the Fermi level from the bulk will be visible. This produces the detected signal close to the Fermi level. There remains one question: if the *E*_*D*_^(0)^ states from the bulk (unaffected by the band bending) manifest in the P^(−)^ case, why do these states not manifest also in P^(+)^ valence band spectra?

One possible answer concerns the spatial extent of the band bending, i.e. the parameter *δ*. In the case of P^(−)^, as stated above (mechanism 2), the compensation charge are ionized donors, which are in large quantity, thus the parameter *δ* is small. For a density of ionized donors near surface corresponding to 0.2 oxygen vacancies per formula unit *N*_*D*_^(+)^ ≈ *N*_*D*_ ≈ 3.2 × 10^27 ^m^−3^, *δ* ≈ *P*/(2*eN*_*D*_) and using *P* ≈ 0.8–1.1 C/m^2^, *δ* yields 0.8–1.1 nm. Using *λ *cos*θ* ≈ 5 Å, the photoemission signal from these levels will be attenuated by about exp(1.6) − exp(2.2) ≈ 5–9, however it will be still visible in the spectrum with a larger intensity than the signal given by the valence band itself, since *N*_*D*_/*N*_*V*_ is at least about 130 for m*(holes) = *m*_*e*_. In the case of P^(+)^, the compensating layer is composed by electrons whose density is much lower, *N*_*C*_ ≈ 6.5 × 10^23^, thus (in a first approximation) the *δ* parameter will be much larger. The complete statistical analysis will be detailed in another work; here we just state that the order of magnitude for *δ* exceeds 10 nm, in agreement with ref. 11. Therefore, the valence band signal from the regions unaffected by the band bending will be attenuated owing to IMFP effects by at least exp(20) ≈ 4.8 × 10^8^. One may also compute that the absolute value of the band bending fits well with the above parameters with a relatively low value of the dielectric constant ∈_*r*_ ≈ 80.

A last comment is needed regarding the width of the individual lines, which is of about 1.1–1.2 eV, including the valence band spectra. This is a rather low resolution for a third generation synchrotron beamline. Indeed, in a recent work using exactly the same setup, C 1s spectra with total width of 0.4 eV were recorded with 400 eV excitation energy, for graphene layers grown on PZT(001) with P^(+)^ orientation^60^. The only explanation of the actual broadening relies, again, on band bending and relatively low values of the parameter *δ* as compared with the IMFP *λ*. Separate contributions with changing binding energies have to be taken into account in an integral over the depth *z*, weighted by exp(−*z*/*λ*). The whole analysis will be detailed in a future work; here we just state that the order of magnitude of the additional broadening is *ePλ*/∈. Introducing *λ* ≈ 7 Å, ∈_*r*_ ≈ 80 and *P* = 1–1.1 C/m^2^ (see below how these values are derived), one obtains an additional broadening of *w*_*a*_ = 1–1.1 eV, which, together with the instrumental broadening *w*_0_ ≈ 0.4 eV gives a total ‘experimental’ broadening on the order of (*w*_*a*_^2^ + *w*_0_^2^)^1/2^ ≈ 1.08–1.17 eV, in line with the observations. Hence, when an appropriate theory will be validated, one may use the line broadening to evaluate the extension of the band bending inside the film (if *P* and *λ* are known, one may estimate the dielectric constant and, from the magnitude of the band bending, one may then estimate *δ*).

### CO desorption with temperature and irradiation with soft X-rays

**Figure 5:**
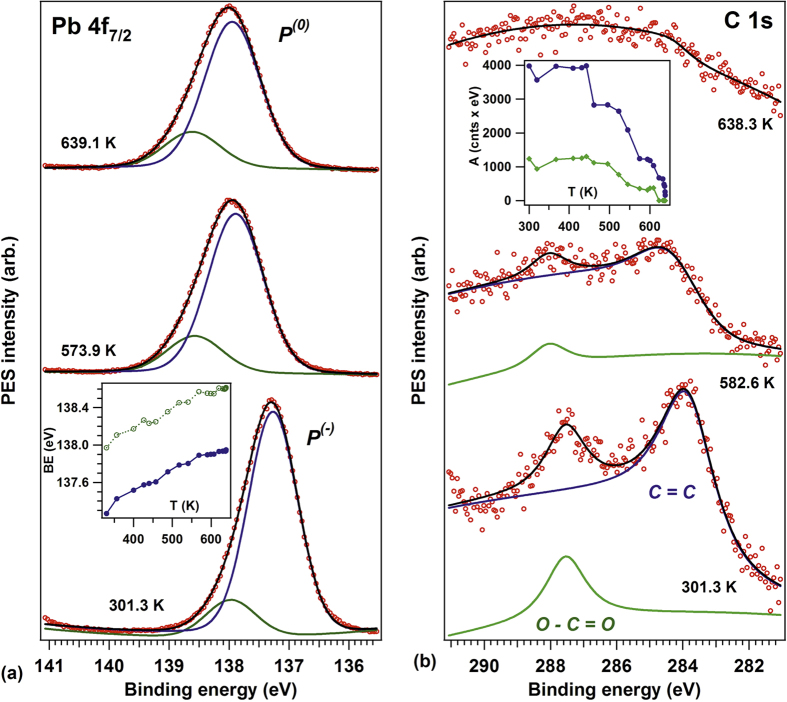
Evolution of Pb 4f_7/2_ (**a**) and C 1s (**b**) core levels with the sample heating, at temperatures indicated, which are averages between the starting and ending temperature for each spectrum. Photon energy: 400 eV in both cases (Pb 4f_7/2_ and C 1s). Vertical offsets are artificially introduced for clarity. Inserted in (**a**) is the evolution of Pb 4f_7/2_ binding energies of the separated components, inserted in (**b**) is the evolution of C 1s integral amplitudes for the separated components. The complete series of spectra is represented in the SI (SI-2). The non-linear baseline in the C 1s spectra (kinetic energy range: 110–120 eV) is attributed to a mixture of Pb Auger and Zr Auger transitions.

Next, we investigate the CO desorption from this surface. The results are presented in Fig. 5, and the complete series of spectra, together with deconvolutions, is presented in SI-2. As stated above, after dosing 6000 L of CO (600 s × 10^−5 ^Torr) on P^(−)^ polarized PZT(001) samples at room temperature, C 1s was visible in the spectra. The C 1s spectra (Fig. 3(b)) are simulated with two peaks, one of lower BE (284.0 eV for the sample before heating, evolves towards 284.5 eV) and another one of higher BE (287.7 → 288.2 eV). These peaks are attributed to graphitic C and to C=O or O-C=O bonds^58^. For the freshly adsorbed CO layer, all peaks are shifted by about 0.7 eV towards lower BE, as expected from the case of a negative band bending by this amount. Heating the sample reduces the band bending according to Fig. 5 (see also SI-2), but, more important, yields to the decrease of the carbon signal.

Consequently, immediately after dosing, the C=C signal corresponds to about one 0.29 ML adsorbed (by considering one ML as one C per PbO surface cell). In the ESI (SI-4) we present a comparison of the C 1s intensity obtained in this case and that obtained for a graphene layer synthesized on Ir(111): the derived carbon coverage (C atoms per PZT(001) surface elementary cell) yields similar values as from the actual considerations implying the use of photoionization cross sections.

By irradiating with soft X-ray photons, both C=C and C=O signals decrease, up to the situation where the [C_*red.*_]:[Pb_*bulk*_] ratio is about 0.25, corresponding to about 1/6 of a ML. Note also that, with irradiation, the ratio between the C=O component and the C=C component decreases from 0.44 to 0.34, therefore a lower amount of carbon is bound to oxygen.

**Figure 6:**
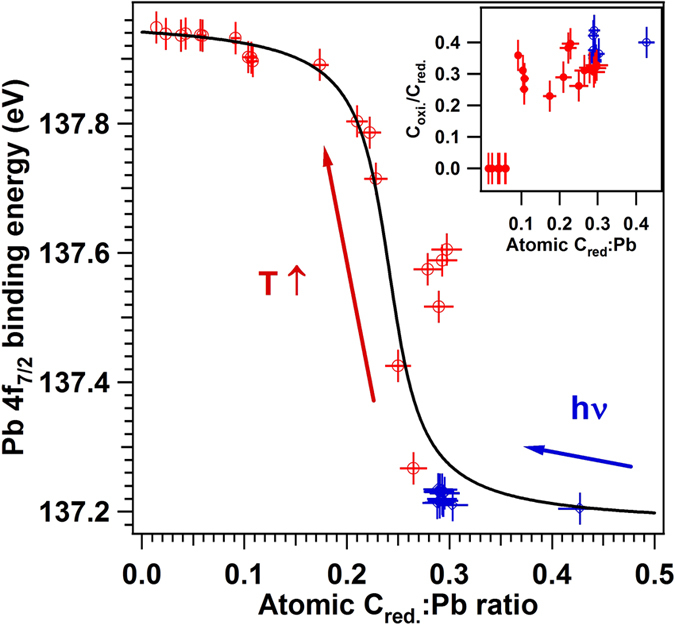
Evolution of Pb 4f_7/2_ BE with the amount of C in C=C state. Inserted upper right: evolution of the ratio between adsorbed oxidized and reduced carbon.

The experimental points from Fig. 6 are fitted with a threshold (tan^−1^) function whose inflection point is found at [C_*red.*_]:[Pb_*bulk*_] ≈ 0.24 ≈ 1/6 ML by taking into account that the ‘visible’ bulk atoms are about 1.5 per surface elementary cell, and its amplitude is found at 0.79 eV. Though affected by the fitting procedure, this value of the band bending between P^(0)^ and P^(−)^ is closer to half of the value derived during the cleaning procedure, when going from P^(+)^ to P^(−)^ (i.e. to 0.72 eV) and to values for band bendings derived in previous experiments on similar samples^12^,^13^,^42^. The fact that the C 1s binding energy (about 284.0 eV) is lower than that of graphitic carbon or the ‘adventitious contamination’ (284.5–284.6 eV) is due to the surface band bending^38^. In fact, if one introduced the derived band bending between the P^(−)^ and the P^(0)^ state of about 0.7–0.8 eV, the C 1s binding energy becomes slightly more elevated (284.7–284.8 eV) than that of the graphitic carbon. Thus, carbon is in a slightly positive ionization state.

One important comment regards the assertion that carbon and CO desorption is really driven by the loss of the polarization P^(−)^ with the increase of temperature. Other thermally activated desorption processes, not necessarily involving the polarization, might be proposed. To support our hypothesis that CO adsorption and desorption is mainly related to the surface polarization state, one has to take into account the following experimental facts: (i) quite few adsorbed C was present when the initial polarization was P^(+)^; (ii) the noticeable decrease of the amount of carbon on the surface from the initial coverage in time, just by irradiating with soft X-rays; (iii) the coincidence of the temperature of vanishing carbon coverage with the temperature where the band bending due to the P^(−)^ polarization vanishes. From metal surfaces or model catalysts, CO desorbs in a relatively wide range of temperatures: 400 K from oxygen-covered Ru(0001)^61^, 500 K from Rh particles on α-Al_2_O_3_(0001)^62^, lower than 400 K for Pd nanoparticles on (1 × 1) TiO_2_(001)^63^, etc. In the actual case, CO sticks on the surface up to more than 600 K (see SI-2) and there are big chances that this stability is intimately related to the polarization state of the PZT layer. A demonstration of this assertion will be detailed in the following.

### Adsorption energy

#### Langmuir model

One considers the Langmuir model for CO adsorption given by the equation:


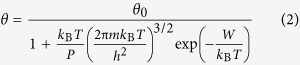


where *θ* is the surface coverage with molecules at temperature *T* and partial pressure *P*, *θ*_0_ the saturation coverage, *k*_B_ the Boltzmann constant, *m* the molecular mass, *h* the Planck constant, and *W* the molecular binding energy on the surface. This equation may be re-written as:


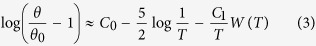


**Figure 7:**
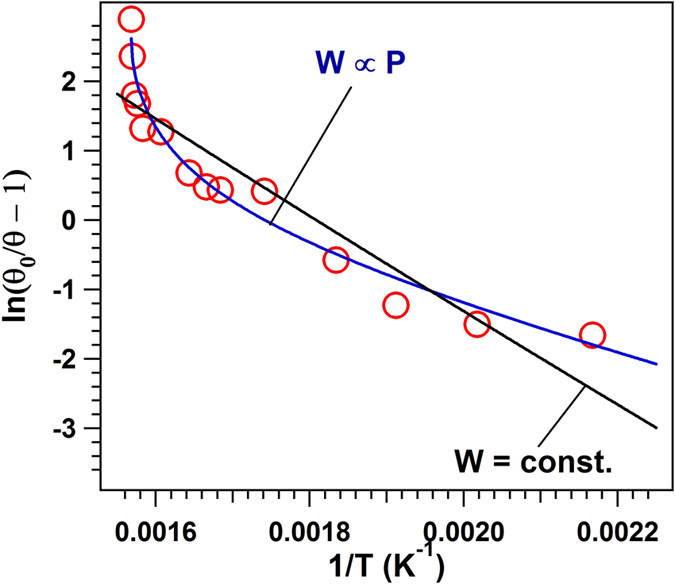
Fitting the Langmuir model, Eq. (3), with constant (black curve) and temperature-dependent (blue curve) adsorption energies, starting from the experimental points represented in Fig. 6 (temperature dependence only).

If the adsorption energy was not temperature dependent, thus any anchoring of molecules involving the substrate polarization dependence on the molecular adsorption would be questionable, since from different determinations, including those plotted in Fig. 3, we know that in the temperature range investigated the polarization is considerably changing. A fit of points plotted such as to exhibit the relation (3) with constant adsorption energy *W* (∂*W*/∂*T* = 0) is not convenient, see Fig. 7. The ideal case is to have an adsorption energy proportional to the polarization *P*(*T*), as it would be yielded by an interaction between the molecular dipole *p* and the field near surface (*W* ≈ *pP*(*T*)/∈_0_). It is argued in ESI (SI-5) that the polarization dependence on temperature may be approximated as *P*(*T*) = *P*_max._{(*T*_*C*_ − *T*)/(*T*_*C*_ − T/3)}^1/2^, where *T*_*C*_ is the Curie temperature. When *T* is close to *T*_*C*_, such dependence is derived also by the Landau theory. If one considers also temperature variations of the dielectric constant (see SI-5), the polarization dependence is slightly more complicated *P*(*T*) ∝ {(*T*_*a*_ − *T*)/(*T*_*b*_ − *T*)}^1/2^. A fit by using the ‘standard’ Langevin theory was not satisfactory, but a good fit was obtained for *W* ∝ {(*T*_*C*_ − *T*)/(*T*_*C*_ − *T*/3)}^1/4^, i.e. the temperature dependence of the adsorption energy is approximately proportional to the square root of the polarization *W*(*T*) ∝ *P*(*T*)^1/2^. We do not have any explanation for such an assertion (binding energy proportional to *P*^1/2^), however we mentioned this effort since it gave a quite fair fit of the data points. By considering the second formula, with two temperature parameters (*T*_*a*_ and *T*_*b*_) into the square root, the fit was also satisfactory and is represented by the blue curve. Hence, the substrate polarization clearly plays a role. Moreover, from (SI-5), the temperatures *T*_*a*_ and *T*_*b*_ may be expressed as:


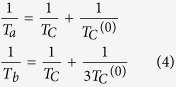


where the *T*_*C*_ parameter enters the expression of the dielectric constant:


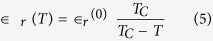


*T*_*C*_^(0)^ is expressed as:


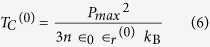


where *n* is the density of dipoles (1/volume of the elementary cell). From the fit of Fig. 7, one obtains *T*_*a*_ = 637.8 ± 1.5 K and *T*_*b*_ = 708.2 ± 21.0 K. From here, *T*_*C*_ ≈ 749.6 K (in good agreement with refs 14 and 15) and *T*_*C*_^(0)^ ≈ 4274 K. Reversing Equation (6), with *P*_max_ = 1.1 C/m^2^ yields a dielectric constant at 0 K ∈_*r*_^(0)^ ≈ 50, which implies ∈_*r*_ ≈ 80 at room temperature. Thus, all these values are consistent with data reported so far on PZT thin films^11^, reinforcing the credibility of the mechanism we proposed for the molecular adsorption, with adsorption energy proportional to the polarization of the substrate.

A variation of the adsorption energy with temperature was proposed also to explain the temperature-programmed desorption of 2-propanol and acetic acid from LiNbO_3_(0001)^5^,^32^,^33^. In these works, the adsorption energy was stipulated to *increase* (linearly) with temperature. The explanation for this hypothesis was sketched in the framework of extrinsic dipole screening of the depolarization field (due to adsorbates). When the polarization of the initially P^(+)^ poled face decreases, there remains excess negative charging at the surface, the surface dipole increases in magnitude, and so does the interaction of adsorbed molecules with these instantaneous surface dipoles. Again, the decrease of the adsorption energy with temperature in the actual case may be seen as another proof for internal screening for initially ultraclean surfaces.

Another discussion may be related to the observed CO dissociation mechanism and to the mechanism of CO adsorption. Molecular carbon monoxide has a quite low dipole moment, of about 0.11 D, oriented from carbon to oxygen^64^. It is easy to compute that the corresponding interaction energy of such a dipole moment in vacuum, in the field of a surface exhibiting 1 C/m^2^ polarization is of about 0.29 eV. However, CO molecules exhibit a rather large polarizability, of about *α*_*V*_ = 1.95 Å^3 ^64. The energy corresponding to the interaction of polarisable molecules may be written as 4π*α*_*V*_*P*^2^/∈_0_ ≈ 17.4 *P*^2^[C/m^2^] eV. This energy seems sufficient to break a large majority of CO molecules. Indeed, the energy for dissociation into ions CO → C^+^ + O^−^ may be computed starting with the ionization energy of CO → CO^+^ + e^−^ (14.01 eV needed^65^) followed by the dissociation of the molecular ion CO^+^ → C^+^ + O (8.33 eV needed^65^) and by the formation of the oxygen anion O + e^−^ → O^−^ (oxygen electron affinity 1.46 eV released^64^), in total 20.88 eV needed, which is the same order of magnitude as the dipole interaction with the surface electric field. It would be sufficient that the surface polarization P ≈ 1.1 C/m^2^ to induce the ionization-dissociation of CO. Through this mechanism, the important dipole moment for molecular adsorption is not the static dipole moment of CO, but the induced moment by the near surface electric field (*p* ≈ 4π*α*_*V*_*P* ≈ 8.1 D for *P* ≈ 1.1 C/m^2^). Next, we consider also the other ionization-dissociation pathway CO → C^−^ + O^+^, where an estimate may be effectuated starting with the dissociation energy of the neutral CO (11.21 eV^65^), adding the ionization energy of neutral oxygen (13.62 eV^64^) and subtract the electron affinity of carbon (1.26 eV^64^), the net energy needed for this path being 23.57 eV. Obviously, the first mechanism, yielding C^+^, is more favourable. Thus, after dissociation, oxygen is rejected by the surface field and pumped away, while C^+^ should attach to an oxygen anion or to a Pb cation from the substrate. Attaching C^+^ to O^−^ induces a local dipole moment oriented outwards, opposed to the orientation of the surface field (inwards), therefore attaching C^+^ to Pb^+^ looks energetically more favourable, since the C–Pb bond is expected to have a dipole moment oriented inwards, from C to Pb (C slightly more negative), thus parallel with the surface field **P**/∈_0_. This should be a basic mechanism to explain why most carbon is found in dissociated state. (The same holds also for (Zr, Ti)O_2_ terminated surfaces, see below). Moreover, the initial positive charge of carbon contributes to the buildup of the hole depolarization charge sheet, reinforcing the surface polarization. We will denote this mechanism as P↓C^+^O^−^, from the orientation of the polarization and the mechanism of dissociation-ionization.

The opposite mechanism, for the same orientation of the polarization (CO → C^−^ + O^+^) or P↓C^−^O^+^, though less favorable energetically, would yield to the attachment of O^+^ to the substrate and the ejection of C^−^, thus no carbon should be visible on the surface. For P^(+)^ polarized surfaces, the mechanism P↑C^+^O^−^ yields rejection of C^+^ ions and attachment of O^−^ ions, while P↑C^−^O^+^ would imply, again, carbon attachment to the surface. Now, it is clear from Fig. 4(a) that this process is less frequent than P↓C^+^O^−^. This is in line with the dissociation energy needed to split the molecule into ions: 20.88 eV for CO → C^+^ + O^−^, achievable with *P* = 1.095 C/m^2^ and 23.57 eV for CO → C^−^ + O^+^, achievable with *P* = 1.16 C/m^2^. Thus, a simple electrostatic evaluation may explain the dissociation of CO on P^(−)^ poled surfaces. It follows also that on P^(+)^ surfaces similar processes may occur, but with only oxygen anions sticking on the surface and carbon cations repelled from it. We may end these considerations by summarizing that the presence of out-of-plane ferroelectric surface polarizes the carbon monoxide up to its dissociation into C^+^ and O^−^, and that C^+^ is adsorbed on P^(−)^ areas only, where finally it sticks on Pb and not on oxygen, in order to form an instantaneous dipole oriented parallel to the field produced by the ferroelectric.

There is no noticeable dependence of the ratio [Pb_*surf.*_]:[Pb_*bulk*_] on the carbon coverage, it rather stays constant around 0.25 ± 0.03. According to the inelastic mean free path estimations from above, the IMFP results as 11.7 Å, which is too elevated for a kinetic energy of about 260 eV. 7–8 Å would be a better value^45^. In this case, the ratio between surface and bulk atoms for a perfect monolayer yields about 0.39–0.45. This implies that the PbO terminated layer covers about 60 ± 4% from the surface. Therefore, some parts of the surface are (Zr, Ti)O_2_ terminated, but in photoemission spectra there is no distinct component corresponding to these surface layers, as is the case of the visible higher BE component of Pb 4f for the surface PbO. One qualitative argument for this is that surface (Zr, Ti) ions have 5 oxygen neighbours, thus the under-coordination with oxygen is 5/6. In the case of surface Pb atoms, the under-coordination is lower, it is 8/12 = 2/3. Thus, if there is a surface shift for surface Zr or Ti atoms, it is not visible within the actual resolution of the spectra from Figs 2(b,c) and 3(b,c). On the other hand, we derived that, after CO dosing, the maximum carbon coverage corresponds to about 29% coverage of the surface. Thus, it may also happen that CO adsorbs mainly on (Zr, Ti)O_2_ terminated surfaces (connected to Zr or Ti to form instantaneous dipoles oriented inwards, as discussed previously) or on steps between (Zr, Ti)O_2_ areas and PbO areas. However, by taking into account the uncertainties in derivation of the IMFPs and also the fact that photoelectron diffraction effects are neglected, the above statement may be regarded just a simple hypothesis to be confirmed by future experiments.

The actual experimental results may be compared, to a limited extent, with recent first principles computations for CO_2_ bonding on PbTiO_3_^66^ (Note that we tried also DFT computations for CO on PZT(001) with different polarizations, but were unable to predict the dissociation of the molecule.). It was found by simulations that on PbO terminated surfaces, the binding energy of CO_2_ in the form of (CO_3_)^2−^ is higher for the P^(−)^ orientation of the polarization than the binding energy of (CO_2_)^2−^ for the P^(+)^ orientation (0.9 eV *vs*. 0.2 eV). Moreover, a metastable dissociated state was proposed for the P^(+)^ case, with CO radicals and O atoms far away from the surface and a quite low binding energy. In the actual work, we obtained experimentally and qualitatively a confirmation of a stronger bonding to the P^(−)^ surfaces than to the P^(+)^ surfaces, despite the different molecule used in our experiment. The proposed mechanism for CO adsorption and desorption involved, at least for C atoms, their stabilization in a slightly positive state, is at variance with all the states proposed for adsorbed molecules in ref. 66, which are in anionic states no matter which is the polarization of the surface. The predicted ability of the P^(+)^ surface to trigger the dissociation at surface deserves a detailed experiment combining XPS and mass spectroscopy. Motivated by the results of the present work where the main mechanism for CO dissociation was attributed to the polarizability of CO, we tried a similar experiment with CO_2_, but were unable to adsorb at room temperature this molecule in detectable quantities; thus, the initial small dipole moment of CO is effective in driving the molecule towards the ferrelectric surface, before the polarization of the molecule starts. It can be estimated that even for the CO dipole moment of 0.11 D, its interaction with the external field of the ferroelectric *P*/∈_0_ yields, for *P* = 1.1 C/m^2^, an interaction energy of about 286 meV, largely exceeding the thermal energy.

Also, recently, binding energies of simple molecules (NO, CO, N_2_, O_2_, NO_2_, CO_2_, SO_2_, H_2_O) on PbTiO_3_ with different out-of-plane polarization, covered by monolayers of RuO_2_ or CrO_2_, were computed by density functional theory (DFT)^67^. The oxide monolayer was chosen for its *a priori* catalytic properties: RuO_2_ is a well known catalyst for NO decomposition, while CrO_2_ was found to be the most effective catalyst of several transition metal oxides in the above numeric study. For CO adsorption, RuO_2_ terminated surfaces did not show any polarization dependence (binding energy of 1.7 eV, constant for P^(+)^, P^(0)^ and P^(−)^), while CrO_2_ terminated surface showed 0.8 eV for P^(+)^, 0.7 eV for P^(0)^ and 0 eV for P^(−)^, thus the strongest bonding was inferred on P^(+)^ faces. The above results are also at variance with the experimental results of this work, where (i) clearly stronger bonding is found for CO on P^(−)^ surfaces at room temperature; (ii) CO adsorbs also in dissociated state, which was not taken into account in ref. 67. One might argue that the systems studied numerically and experimentally are not exactly the same, by the presence of the oxide monolayer in the numeric study, while the experimental study concentrated on Pb(Zr, Ti)O_3_ and not on PbTiO_3_, therefore a ferroelectric with larger values of polarizations. Such discrepancies are expected to stimulate further studies, both theoretical and experimental. In this paragraph we discussed adsorption mechanisms in terms of a very simple electrostatic model, with confidence given by the ability to fit the desorption curves with an adsorption energy whose temperature dependence is close to that of the polarization.

### Oxygen depletion

**Figure 8:**
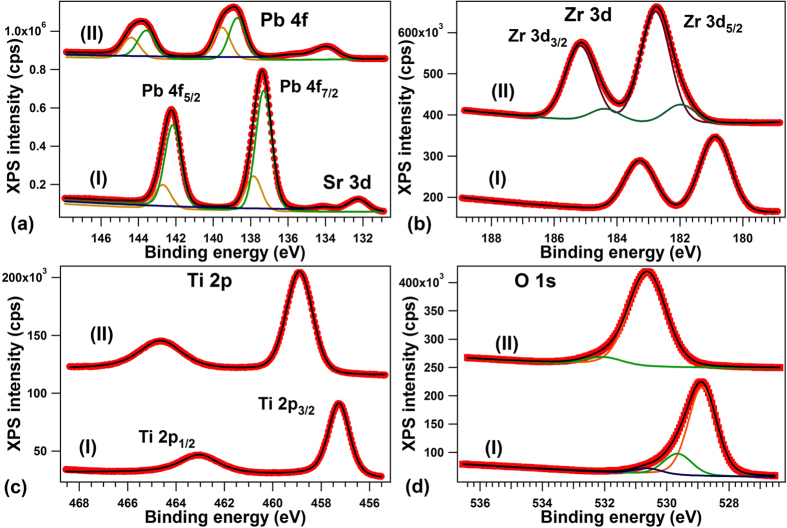
Evolution of (**a**) Pb 4f, (**b**) Zr 3d, (**c**) Ti 2p, (**d**) O 1s spectra for freshly prepared P^(−)^ poled PZT(001)–curves (I) and after two cycles of CO adsorption → thermally induced desorption–curves (II). Photon energies are the same as in Figs 2, 3 and 4.

The last question which will be addressed concerns the way carbon is desorbed from the surface. Figure 8 presents XPS spectra obtained in similar conditions as those presented in Figs 2, 3 and 4, for a clean PZT(001) sample, without C contamination and exhibiting P^(−)^ polarization, and the spectra obtained on the same surface after two cycles of CO adsorption and desorption, as commented in the previous Subsections. The Pb 4f integral amplitude decreases to 0.52 of its initial value, whereas the Ti 2p increases by a factor 1.50, Zr 3d increases by a factor 1.57, and O 1s increases by a smaller amount, a factor 1.16. The immediate suggestion of these intensity variation is that the layer changes its termination from PbO to (Zr, Ti)O_2_. By taking into account IMFP effects (and assuming that the IMFP, *λ*, is the same for all core levels, putting also *λ*′ = *λ* cos *θ*) and noting by *I*_0_ the intensity corresponding to one atom per surface elementary cell and by *c* the out-of-plane lattice constant, for PbO terminated films the XPS intensity may be written as:


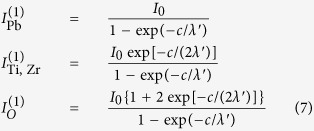


while for (Ti, Zr)O_2_ terminated films the formulas are:


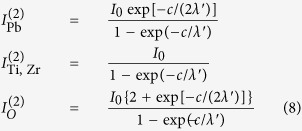


It follows that the ratio between the intensities from (Ti, Zr)O_2_ termination and PbO termination should be exp{−*c*/(2*λ*′)} for Pb, exp{*c*/(2*λ*′)} for Ti and Zr and 1 + {exp[−*c*/(2*λ*′)] + 1}^−1^ for oxygen. The above numbers yield a slight Pb depletion (by 0.20 ± 0.02 per formula unit), but the most important is the oxygen depletion. From the Ti and Zr ratios (yielding a coherent value of *λ*′ = *λ* cos*θ* ≈ 4.81 ± 0.25 Å), the oxygen ratios (*I*_O_^(2)^/*I*_O_^(1)^) should have yielded between 1.61 and 1.67, while the obtained experimental ratio is 1.16. Thus, the surface is not only (Ti, Zr)O_2_ terminated, but also oxygen depleted, with a surface composition (note that the IMFP is quite low) which may be computed, in a first approximation, as Pb(Ti, Zr)O_2.12±0.04_.

By inspecting the second adsorption-desorption process (not shown), we estimate that the carbon amount adsorption during this process was about one half of the first one, i.e. roughly 0.15 ML. Thus, after two cycles of about 0.29 + 0.15 = 0.44 carbons per surface elementary cell adsorbed, the desorbed species are one Pb atom and about 1.88 oxygen atoms. These oxygen atoms are most likely desorbed in the form of PbO + 0.44 CO_2_. Of course, this is just a first evaluation based on IMFP considerations; again, photoelectron diffraction effects are neglected and the IMFP was considered the same for all core levels investigated.

We may then end this section by completing the adsorption – thermal desorption picture for CO and proposing that, despite the fact that CO is in greater amount dissociated when it is adsorbed on PZT(001), once the polarization of the substrate vanishes, carbon is desorbed by uptaking oxygen from the substrate, most probably in the form of CO_2_.

Finally, we derive from Fig. 8 that, after these two desorptions, the P^(+)^ polarization is recovered, by a strong shift towards higher BE for all core levels (1.68 eV for Pb, 1.90 eV for Zr, 1.63 eV for Ti, and 1.77 eV for oxygen). The presence of a strong concentration of oxygen vacancies provide electrons which may be used to screen the depolarization field inside the layer, as discussed in greater detail in refs 10,11,44. After both desorption cycles, one may estimate that only the 0.88 oxygen vacancies (per surface elementary cell of area *a*^2^) from the first two layers provide a mobile charge able to compensate the depolarization field produced by a polarization of about 0.93 C/m^2^ (if one considers that each oxygen vacancy contributes with one electron to the charge carrier density and that the surface lattice constant is taken as *a* ≈ 3.9 Å).

## Conclusions

This work proved evidence for P^(−)^ state of atomically clean PZT(001), that one can follow the polarization state during thermal treatments by simply watching the band bending, and that CO adsorption at room temperature saturates in the sub-monolayer regime, exhibiting mostly dissociated CO. A fast desorption occurs when the layers are irradiated with soft X-rays, and the desorption dependence on the temperature allows one to infer that sticking of CO on PZT is intimately connected to the surface P^(−)^ polarization state. The fact that no more CO may be adsorbed at room temperature after saturating the surface by one C (or CO) for 3–4 surface elementary cells of PZT(001) is also an interesting result and for sure will stimulate the interest for *ab initio* calculations. In this experimental paper we presented basic arguments for an electrostatic model in which molecules are polarized by the substrate, and subsequently they achieve a dipole moment so important that the interaction energy with the electric field of the surface suffices to dissociate the molecule into separate ions. C^+^ is attracted to the surface, while O^−^ is repelled. The temperature dependence of the adsorption energy was found to be proportional to that of the film polarization, in a modified model where one considers also the variation of the dielectric constant with temperature. These simple experimental assertions should serve to stimulate a deeper theoretical work.

In order to explain the observed preferential CO adsorption on the P^(−)^ areas, an important information was offered by the valence band measurements, which are for the first time in this work interpreted in a different way: the onset of the photoemission signal for P^(+)^ areas does not correspond to photoemission from the valence band, but to photoemission from the shallow donor levels (oxygen vacancies). The density of oxygen vacancies for P^(+)^ areas is by several orders of magnitude higher than the density of electronic states in the conduction band. Finally, the desorption of the dissociated carbon proceeds by uptaking oxygen from the PZT layer, and most probably carbon is desorbed in form of CO_2_. Upon absorption-desorption processes, PbO layers from the surface are also desorbed. When sufficient oxygen depletion is achieved, the films return to the P^(+)^ state.

Consequently, inwards polarized PZT surfaces are good candidates for both CO breakdown and subsequent oxidation, by using oxygen from the film, which may be recovered by subsequent thermal treatment. The only drawback of the actual data is the slight PbO depletion during annealing; we hope that controlled annealing at lower temperatures might induce a smaller amount of Pb eliminated from the sample; however, for practical applications in the field of environmental technology, one needs to continue this work on Pb-free ferroelectrics, such as BaTiO_3_ or alkali niobates.

## Methods

PLD preparation procedures of PZT(001) thin layers on SrTiO_3_(001) single crystals with 20 nm SrRuO_3_ buffer layers are described in greater detail in previous works^12^,^13^,^36^. A KrF laser is used (wavelength 248 nm, repetition rate 5 Hz, pulse 0.7 J × 20 ns, laser fluence 2 J/cm^2^), the oxygen partial pressure is 20 Pa and the substrate is held at 850 K during deposition. Under these conditions, outwards polarization is expected to occur^50^.

Piezoresponse force microscopy (PFM) is achieved by using an MFP-3D Asylum Research setup. Photoelectron spectroscopy was performed using the SuperESCA beamline at the Elettra light source in Trieste, Italy (source: undulator 21.28, monochromator: SX700, estimated flux: 10^12^ photons/s at the sample). The experimental station comprises a load-lock, a preparation chamber and an analysis chamber equipped also with low energy electron diffraction (LEED) optics, both ultrahigh vacuum (UHV) chambers operating at a base pressure of 1 × 10^−10 ^mbar. The spot size on the sample of the synchrotron light is 100 × 10 μm (*w* × *h*). Electrons are analyzed at 40° takeoff angle by using a 150 mm mean radius (Specs Phoibos) electron energy analyzer operating in medium area mode with pass energy of 10 or 20 eV (the latter pass energy was used for C 1s spectra only, due to their lower intensity). Careful intensity calibration was performed between spectra recorded with different pass energies (a factor or 2.8 occurs between C 1s spectra measured with 400 eV photon energy at 20 eV and 10 eV pass energy, respectively) and between spectra recorded at different photon energies. Binding energies are calibrated with respect to the Fermi level measured on an Au foil mounted close to the sample surface, or to separate experiments on Ir(111) or Pt(001). Sample annealing is achieved resistively with a filament, and temperature regulation is possible by using a Eurotherm regulator; the temperature is measured close to the sample surface. Oxygen and CO are introduced from a well outgassed UHV gas line, from ultrapure gas cylinders. After annealing 3–4 hours in O_2_, PZT(001) layers exhibited reasonable LEED patterns, as represented in Fig. 2(f). LEED *I*-*V* curves were also recorded, but will be presented in a subsequent work.

## Additional Information

**How to cite this article**: Tănase, L. C. *et al.* Ferroelectric triggering of carbon monoxide adsorption on lead zirco-titanate (001) surfaces. *Sci. Rep.* **6**, 35301; doi: 10.1038/srep35301 (2016).

## Supplementary Material

Supplementary Information

## Figures and Tables

**Table 1:** Results of the intensity analysis of core level spectra.

Sample	State	[Pb_*bulk*_]: [ZT*]	[Zr]: [ZT*]	[O]:[ZT*]	[Pb_*surf.*_]: [Pb_*bulk*_]	[Sr]: [Pb_tot._^**^]
#1	as introduced	0.68	0.18	1.87	0.263	1.20%
	1^st^ annealing	1.21	0.20	2.29	0.456	1.23%
	2^nd^ annealing	1.51	0.22	2.99	0.326	1.50%
#2	annealed, before heating	1.45	0.23	3.46	0.355	—
	after heating	1.38	0.24	3.35	0.407	—
#3	annealed, before CO	1.61	0.24	2.97	0.362	1.44%

The ratio are computed from integral intensities of photoemission spectra, normalized with respect to the atomic photoionization cross sections^54^ and to the soft X-ray flux of the beamline. Photoelectron diffraction effects are not taken into account.

^*^ZT = Zr + Ti.

^**^Pb_tot._ = Pb_surf._ + Pb_bulk_.
