# Supplementary material for: Ferroelectric triggering of carbon monoxide adsorption on lead zirco-titanate (001) surfaces
Source: Sci Rep. 2016 Oct 14;6:35301. doi: 10.1038/srep35301 (PMC5064406; doi:10.1038/srep35301)
Supplement: Supplementary Information [file srep35301-s1.doc]

Supplementary Information

**Ferroelectric triggering of carbon monoxide adsorption on lead zirco-titanate (001) surfaces**

L. C. Tănase^a,b^, N.G. Apostol^a^, L.E. Abramiuc^a,b^, C.A. Tache^a,c^, L. Hrib^a^, L. Trupină^a^, L. Pintilie^a^ and C. M. Teodorescu^a,†^

a. National Institute of Materials Physics, Atomiştilor 105b, 077125 Măgurele-Ilfov, Romania

b. University of Bucharest, Faculty of Physics, Atomiştilor 405, 077125 Măgurele-Ilfov, Romania

c. University of Trieste, Department of Physics, Via Valerio 2 - 34127 Trieste Italy

^†^Corresponding Author, e-mail: [teodorescu@infim.ro](mailto:teodorescu@infim.ro)

**SI-1.** **Discussion of binding energies (in eV) and of sample polarization from the actual work and from previous publications dealing with samples prepared in similar conditions (only main components listed from the deconvolutions).** Estimated errors: ± 0.02 eV.

| PZT(001) sample | Polarization | Pb 4f_7/2_ | Zr 3d_5/2_ | Ti 2p_3/2_ | O 1s |
| --- | --- | --- | --- | --- | --- |
| this work, 20 nm, as introduced | P^(+)^ | 138.91 | 182.30 | 458.70 | 530.37 |
| this work, 20 nm, 1st annealing in O_2_ | P^(+)^ | 138.72 | 182.33 | 458.60 | 530.30 |
| this work, 20 nm, 2nd annealing in O_2_ | P^(-)^ | 137.25 | 180.80 | 457.20 | 528.82 |
| this work, 20 nm, second preparation | P^(-)^ | 137.32 | 180.89 | 457.26 | 528.80 |
| Ref. [12], 150 nm, no annealing | *P^(+)^* | *137.60*  *138.50* | *181.05*  *181.95* | *457.60*  *458.50* | *528.95*  *529.85* |
| Ref. [36], 150 nm, fresh, as introduced | P^(+)^ and P^(-)^ | 138.55 | 181.80 | 457.02 | 527.74 |
| Ref. [36], 150 nm, annealed in UHV | P^(+)^ | 139.06 | 181.48 | 458.18 | 529.73 |

Note that in Ref. [12] all spectra were re-calibrated in binding energies to the inherent contamination peak of C 1s, attributed to 284.6 eV. After more experiments on such kind of samples, it turned out that contaminants, when adsorbed on P^(+)^ areas, exhibit core levels shifted by about 0.9 eV towards higher binding energies, featuring the term *ePδ*/∈, see the main text and also Ref. [13]. This was evidenced in Ref. [36], where freshly prepared samples exhibited low binding energies, whereas the core levels of the contaminants were shifted towards higher binding energies. Applying these corrections to the data from Ref. [12], one obtains with a good approximation binding energies in line with the other experiments where the P^(+)^ state was inferred also by other methods (PFM). These corrected data are represented with red color. Therefore, it is reasonable to assume that in the actual work the initial polarization state is P^(+)^, which is kept after the first annealing in O_2_ and only after the second annealing in O_2_ the P^(-)^ state is induced. The agreement between the binding energy vales from the Table below is remarkable if one takes into account that the experiments were performed in three different setups, by using conventional X-ray sources (previous work) or synchrotron radiation with different photon energies (actual work).

**SI-2.** **Complete series of Pb 4f and C 1s spectra as function on temperature**


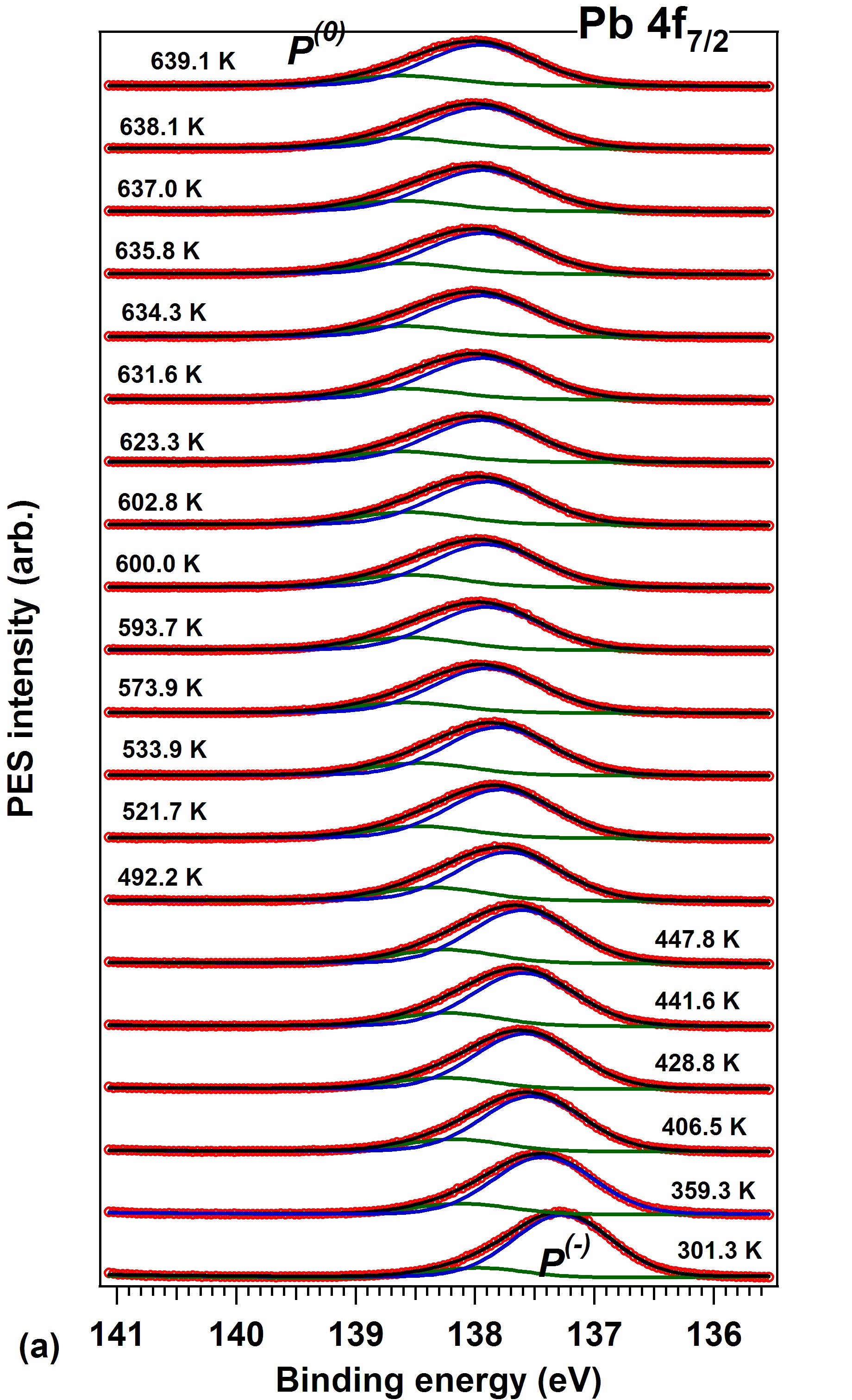

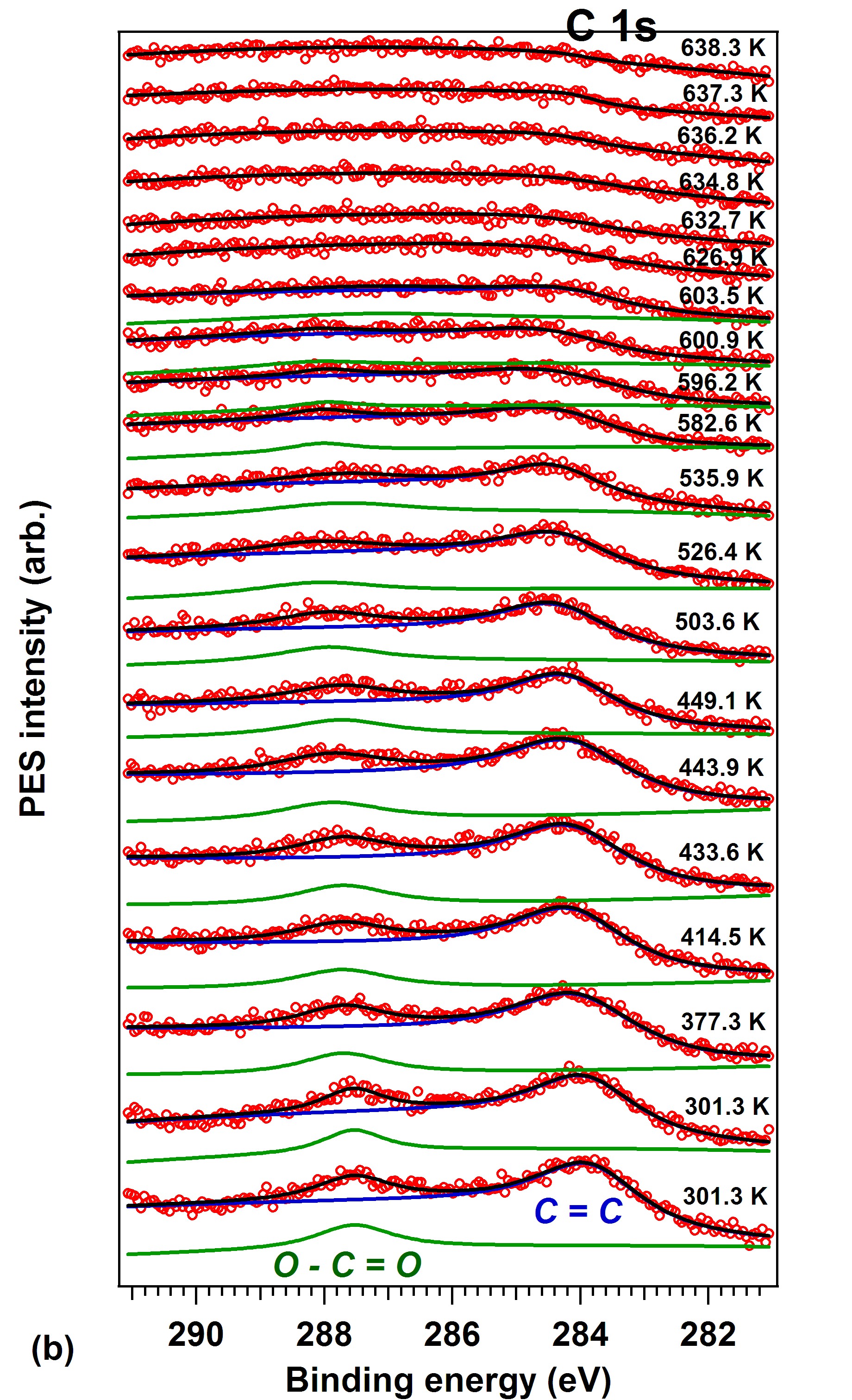


Figure SI.1. Evolution of Pb 4f (a) and C 1s (b) core levels with the sample heating, at temperatures indicated, which are averages between the starting and ending temperature for each spectrum. Photon energy: 400 eV. Vertical offsets are artificially introduced for clarity.

**SI-3.** **Evolution of Pb 4f_7/2_ and C 1s core levels for CO/PbZr_0.2_Ti_0.8_O_3_(001) as function on the irradiation time with soft X-rays.**

| 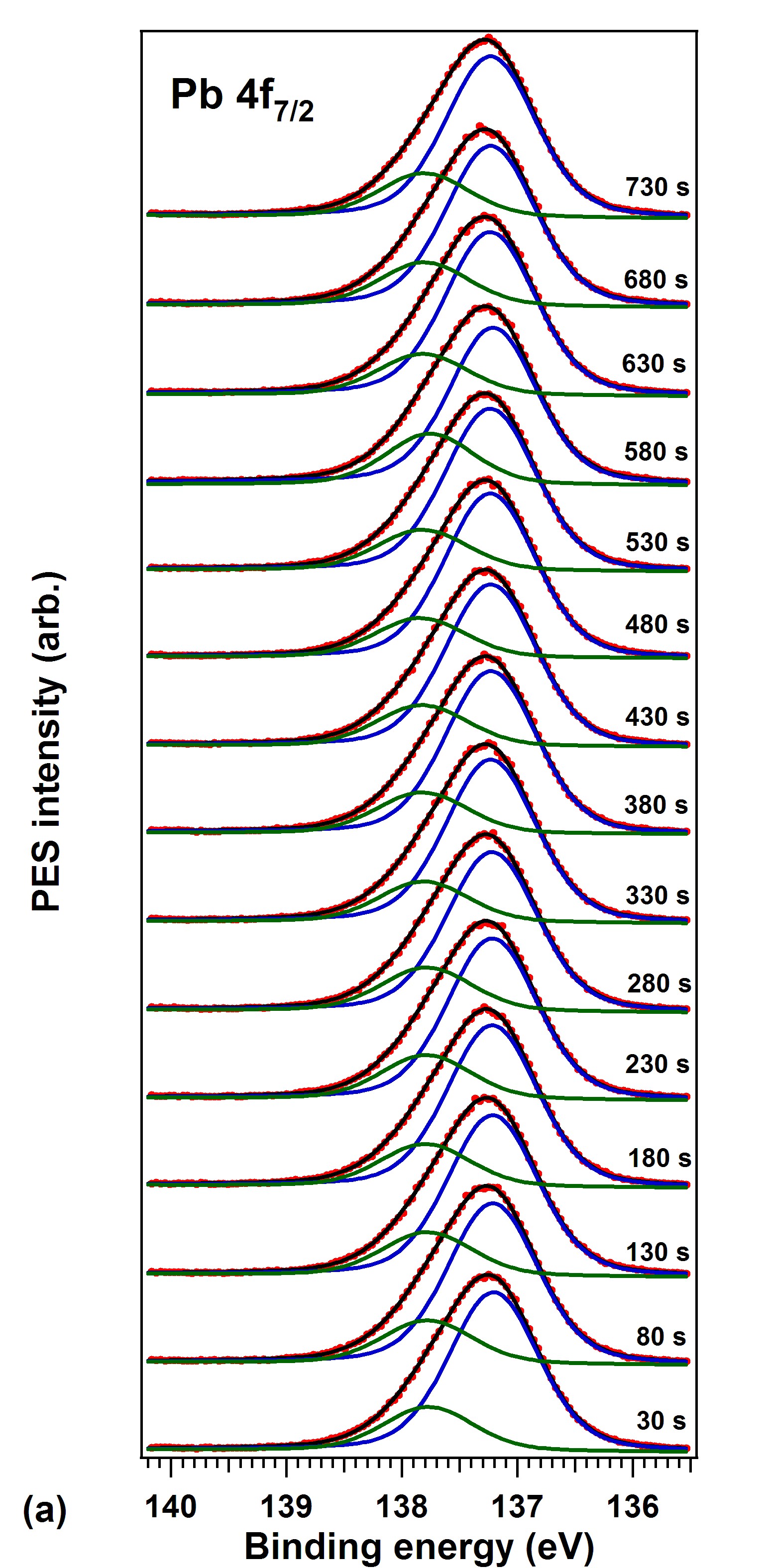 | 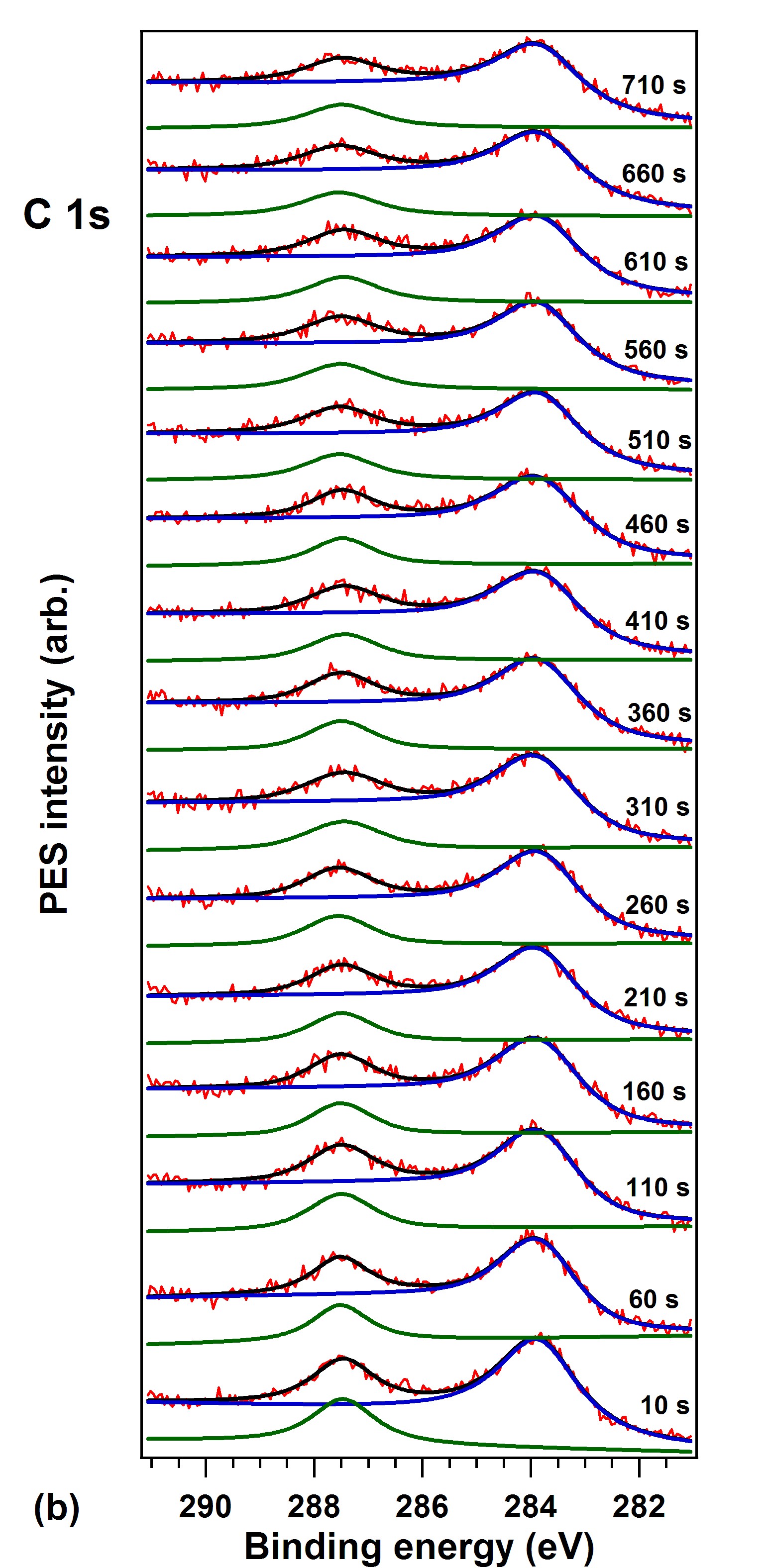 |
| --- | --- |

Figure SI.2. (a) Pb 4f_7/2_ and (b) C 1s evolution with irradiation by soft X-rays at 400 eV photon energy, starting with the clean PZT(001) surface after exposure at 6000 L of CO. The time at the middle of each spectrum is indicated. Vertical offsets are artificial.

**SI-4.** **Comparison of C 1s XPS intensity between saturated CO/PZT(001) and 1 ML graphene synthesized on Ir(111), measured in similar conditions.**


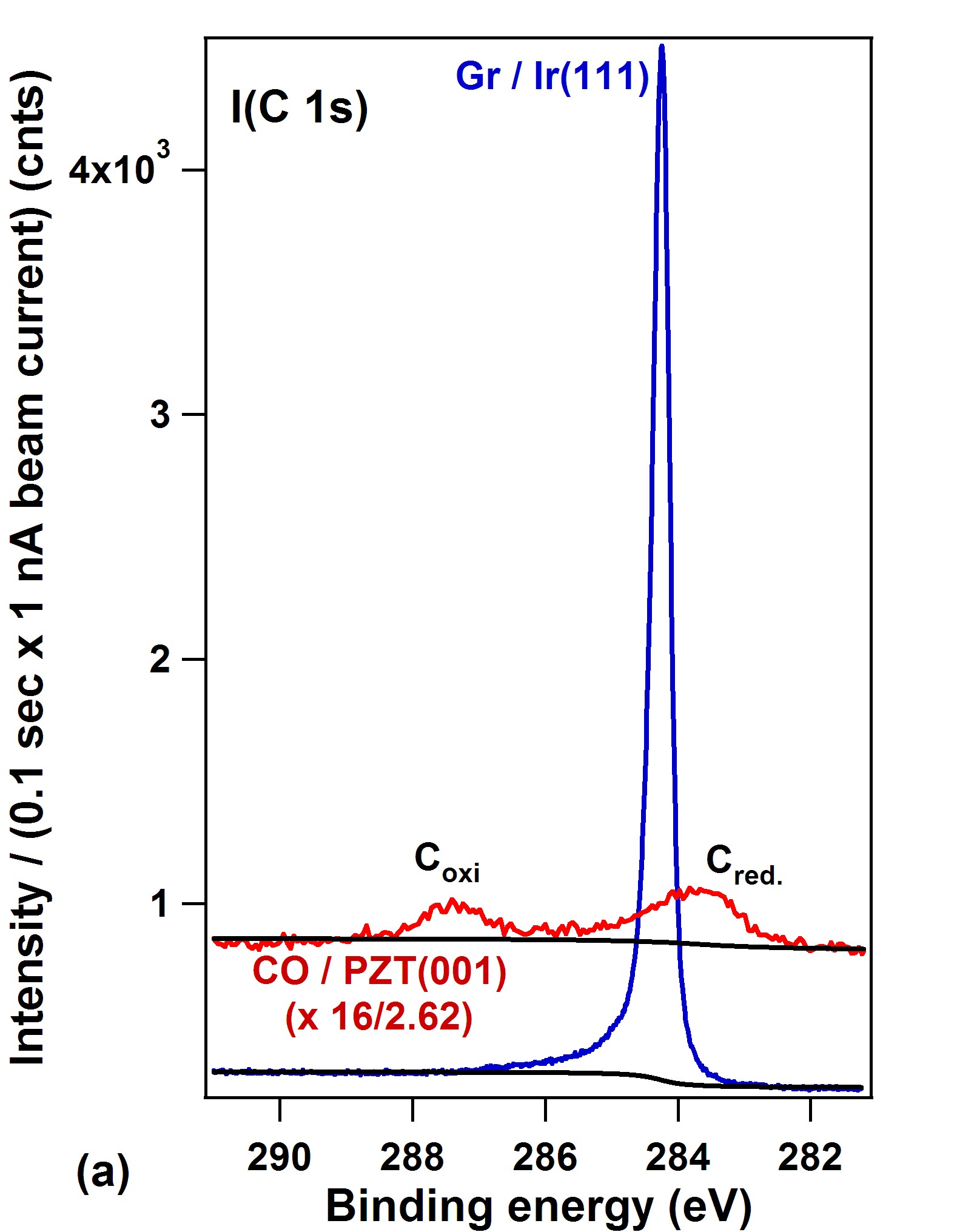

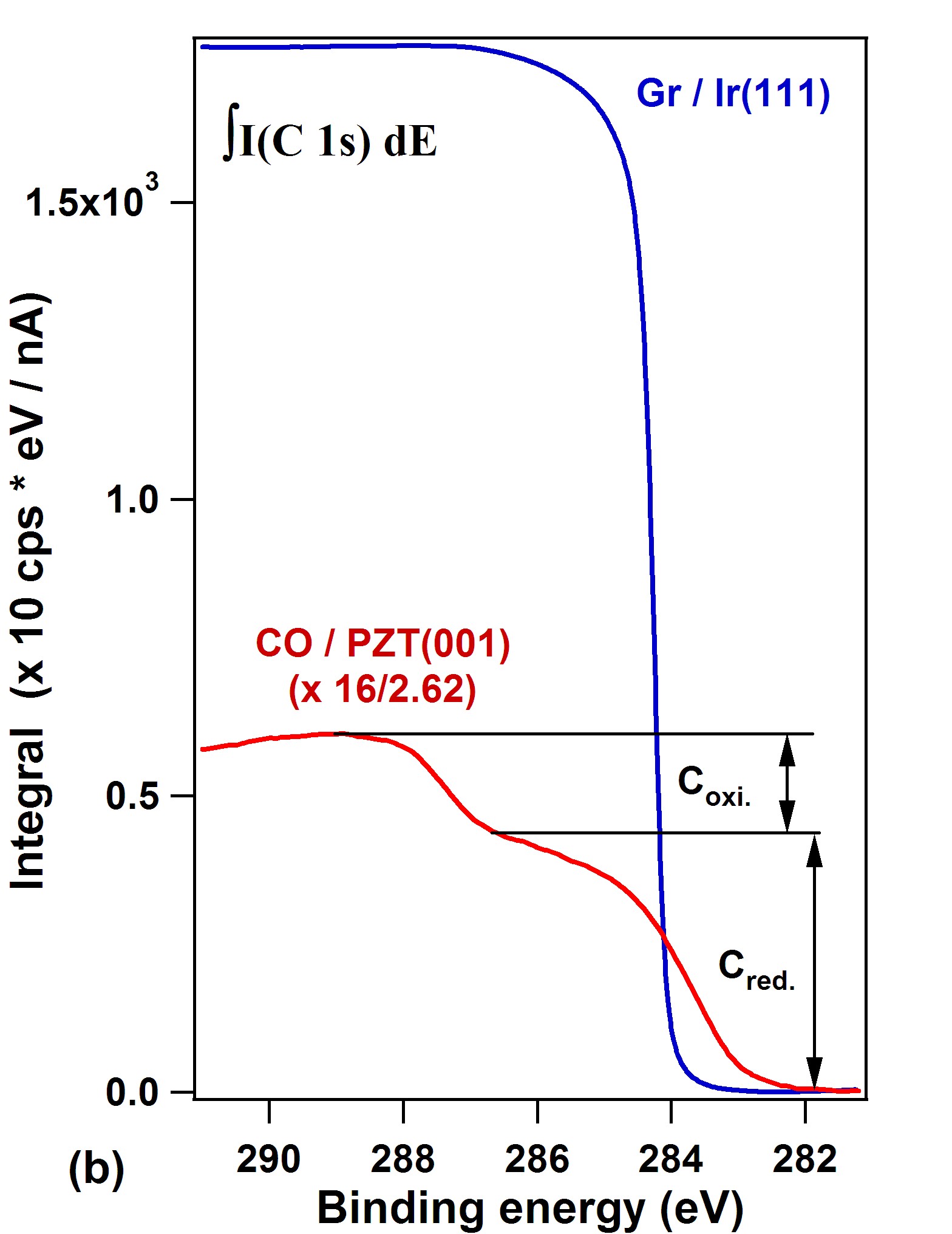


Figure SI.3. Comparison of intensities between a freshly CO dosed PZT(001) surface and a graphene single layer grown on Ir(111): (a) XPS spectra; (b) Integrals of XPS spectra, after background subtraction.

The spectrum of CO/PZT(001) was multiplied by a factor 16/2.62, 16 Å^2^ representing approximately the area of the surface elementary cell of PZT(001), while 2.62 Å^2^ is the area corresponding to one C atom for graphene (3*a*^2^√3/4, with *a* = 1.42 Å the C-C nearest neighbor distance in graphene). In this way, the ratio between the integral of the CO/PZT spectrum and that of the graphene spectrum may be used to derive the C coverage in terms of carbon atoms per surface units cell of PZT. Prior to integration, from the spectra we subtracted a Shirley background function (a double Shirley background for CO, since in this case there are two components). The result is that the total amount of carbon represents about 0.34 ML, the reduced carbon is of about 0.25 ML, and the oxidized carbon is one C atom for about 0.1 ML. These results are in good agreement with the findings of § 3.5., by using [C]:[Pb] ratios, IMFP considerations and theoretical photoionization cross sections.

**SI-5. Polarization dependence with temperature, Langevin model**

Here we will adapt the Langevin theory of ferromagnetism for ferroelectricity [SI1]. The interaction of a dipole *p* with an electric field *E* is *H* = – *p*∙*E*. In the one-dimensional problem, with the magnitude of p ranging from – *p*_max_ to *p*_max_, the statistical average, with *β* = (*k*_B_*T*)^-1^ is given by:

$\begin{matrix} \left\langle p\right\rangle=\frac{\int_{-p_{\max}}^{p_{\max}} p\exp\left(\beta pE\right)dp}{\int_{-p_{\max}}^{p_{\max}} \exp\left(\beta pE\right)dp}=\frac{\partial}{\partial\left(\beta E\right)}\ln\int_{-p_{\max}}^{p_{\max}} \exp\left(\beta pE\right)dp \\ =p_{\max}\left\{\coth\left(\beta p_{\max}E\right)-\frac{1}{\beta p_{\max}E}\right\}\equiv p_{\max}L\left(\beta p_{\max}E\right) \end{matrix}$ (SI.1)

where *L*(*x*) is the Langevin function. In the presence of the polarization *P*, the internal field is *P*/∈, ∈ being the permittivity of the crystal, assumed constant. We note *u* = 〈*p*〉/*p*_max_ and *P* = *n*〈*p*〉 = *np*_max_*u*, *n* being the density of dipoles. Moreover, with

$a=\frac{p_{\max}^{2}n}{\in k_{\text{B}}T}=\frac{P_{\max}^{2}}{\in nk_{\text{B}}T}$ (SI.2)

P_max_ being the maximum (saturation) polarization, ferroelectricity occurs when the equation:

$u=L(au)$ or $au=L^{-1}(u)$ (SI.3)

has a non-vanishig solution. The inverse of the Langevin function *L*^-1^ accepts the following analytical approximation [SI1, SI2]:

$L^{-1}(u)\approx\frac{3u-u^{3}}{1-u^{2}}$ (SI.4)

This has analytical solutions:

$u=\pm u_{r}=\pm\left(\frac{a-3}{a-1}\right)^{1/2}$ (SI.5)

where *u_r_* are (reduced) values of the remanent polarization. Valid solutions are found for *a* ≥ 3, hence the Curie temperature may be computed as:

$T_{C}=\frac{P_{\max}^{2}}{3\in nk_{\text{B}}}$ (SI.6)

and *a* = 3*T_C_*/*T*. For *P* = 1 C/m^2^, ∈*_r_* = 250 and *n* =1/64 Å^-3^, one computes *T_C_* ≈ 702 K.

From eq. (SI.5), the remanent polarization may be expressed as:

$P_{r}=P_{\max}\left(\frac{T_{C}-T}{T_{C}-T/3}\right)^{1/2}\equiv P_{\max}\left(\frac{1/T-1/T_{C}}{1/T-1(3T_{C})}\right)^{\beta}$ (SI.7)

where *β* = 1/2 is the Landau critical exponent.

If the dielectric constant varies with the temperature in the ferroelectric state, such as [SI3]:

$\in_{r}(T)=\in_{r}^{(0)}\frac{T{}_{C}}{T_{C}-T}$ (SI.8)

introducing this in equation (SI.2), then in (SI.5) yields *a* = 3*T_C_*^(0)^(*T*^-1^ – *T_C_*^-1^), where:

$T_{C}^{(0)}=\frac{P_{\max}^{2}}{3n\in_{0}\in_{r}^{(0)}k_{\text{B}}}$ (SI.9)

and finally

$P_{r}\propto\left\{\frac{\frac{1}{T}-\left(\frac{1}{T_{C}}+\frac{1}{T_{C}^{(0)}}\right)}{\frac{1}{T}-\left(\frac{1}{T_{C}}+\frac{1}{3T_{C}^{(0)}}\right)}\right\}^{1/2}\propto\left(\frac{T_{a}-T}{T_{b}-T}\right)^{1/2}$ (SI.10)

and from here follow equations (4) used in the main text.

**References:**

[SI1] Lungu, G.A.; Apostol, N.G.; Teodorescu, C.M. *Basic concepts in ferromagnetism of diluted magnetic semiconductors. The case of manganese embedded in Ge(001)*, in *Nanomagnetism*, J.M. Gonzalez Estevez (Ed.), OneCentralPress, Manchester 2014, pp. 74-110, ISBN: 978-1-910086-05-6.

[SI2] Cohen A. A Padé approximant to the inverse Langevin function. *Rheologica Acta* 1991, *30*, 270-273.

[SI3] Yurtseven, H.; Kiraci, A. Temperature dependence of the polarization and the dielectric constant near the paraelectric-ferroelectric transitions in BaTiO^3^, *J. Mol. Model.* 2013, *19*, 3925–3930.
